# Gene biomarker discovery at different stages of Alzheimer using gene co-expression network approach

**DOI:** 10.1038/s41598-020-69249-8

**Published:** 2020-07-22

**Authors:** Negar Sadat Soleimani Zakeri, Saeid Pashazadeh, Habib MotieGhader

**Affiliations:** 1 Faculty of Electrical and Computer Engineering, University of Tabriz, Tabriz, Iran; 2 Department of Computer Engineering, Gowgan Educational Center, Tabriz Branch, Islamic Azad University, Tabriz, Iran

**Keywords:** Biomarkers, Biomarkers, Genetics research, Systems biology

## Abstract

Alzheimer's disease (AD) is a chronic neurodegenerative disorder. It is the most common type of dementia that has remained as an incurable disease in the world, which destroys the brain cells irreversibly. In this study, a systems biology approach was adopted to discover novel micro-RNA and gene-based biomarkers of the diagnosis of Alzheimer's disease. The gene expression data from three AD stages (Normal, Mild Cognitive Impairment, and Alzheimer) were used to reconstruct co-expression networks. After preprocessing and normalization, Weighted Gene Co-Expression Network Analysis (WGCNA) was used on a total of 329 samples, including 145 samples of Alzheimer stage, 80 samples of Mild Cognitive Impairment (MCI) stage, and 104 samples of the Normal stage. Next, three gene-miRNA bipartite networks were reconstructed by comparing the changes in module groups. Then, the functional enrichment analyses of extracted genes of three bipartite networks and miRNAs were done, respectively. Finally, a detailed analysis of the authentic studies was performed to discuss the obtained biomarkers. The outcomes addressed proposed novel genes, including MBOAT1, ARMC7, RABL2B, HNRNPUL1, LAMTOR1, PLAGL2, CREBRF, LCOR, and MRI1and novel miRNAs comprising miR-615-3p, miR-4722-5p, miR-4768-3p, miR-1827, miR-940 and miR-30b-3p which were related to AD. These biomarkers were proposed to be related to AD for the first time and should be examined in future clinical studies.

## Introduction

Alzheimer is an incurable neurological disorder and is classified as an aging disease. It is one of the important neurological complications which can affect the whole society ranging from the patients themselves to the people who are around them. The aging population is growing in many countries, and the treatment costs of Alzheimer are dramatically high. These issues have drawn the attention of many researchers to the importance of the examination of this disease^1^. There are many organizations all over the world which work in the field of early diagnosis and prevention of Alzheimer^2,3^. National center for health statistics considers Alzheimer's disease as the sixth cause of death in the United States^4^. As a result, Alzheimer's disease is among the costliest diseases for various socioeconomic classes. As the population of the world grows, the number of inflicted people increases. Therefore, the control of the affected population becomes more difficult^5^. Significant advances in medical and neurological sciences have led to a longer life expectancy and have increased the number of Alzheimer's disease patients. Ultimately, the prevention of disease before its occurrence is regarded to be one of the most important pillars of treatment at different stages of this disease. Treatment or postponement of a disease depends on its discovery by identifying the biological pathways involved in the disease and adopting various drug-disease network approaches^6^ to control these pathways.

In recent decades, deep investigation of molecular mechanisms has become more prevalent as a research method for finding effective treatments for complex diseases such as cancer, diabetes, Alzheimer, and so on. Microarray and Next-Generation Sequencing (NGS) are the most common technologies utilized in the relevant research studies^7^. Related studies that use deep investigation methods focus on the preprocessing stage, which involves the different feature selection or dimension reduction algorithms^7,8^. Almost all of the previous studies in this field can be classified into two main categories. The studies in the first category have adopted image processing approaches based on brain images (e.g., MRI)^9–17^.

On the other hand, the studies in the second category have used gene expression data to predict the chance of developing Alzheimer’s disease^1,6,18–28^. An article that used Linear discriminant analysis as the best separation procedure is an example of the studies which fall into the first category. It used pathway analysis to distinguish between different stages of Alzheimer. More specifically, it classified different stages of Alzheimer’s disease using pathway analysis^29^. The investigation of meta-analysis studies and the examination of the studies which fall into these two categories highlight a significant gap in the relevant literature on Alzheimer’s disease and reveal that there is a need for further research on this disease.

A brief description of weighted gene co-expression network analysis (WGCNA) and some of the related studies which have adopted this approach is necessary to clarify our method. The WGCNA describes patterns that are constructed as a result of the correlation between the genes in microarray data. It is one of the system biology methods and is used in this study. It is a very useful R package that can be used to construct gene co-expression networks or to discover modules and correlations between genes. Moreover, it can also be utilized to identify Eigen genes or intra-modular hub genes or to calculate measurement values for the module memberships and topological properties^30^. A study adopted this method and used gene and miRNA expression data to discover some diagnostic biomarkers for the early detection of Colorectal Cancer. First, it utilized clustering to extract low preserved modules by constructing the co-expression networks for the different stages of colorectal cancer. Second, it reported two novel miRNAs that were related to colorectal cancer as biomarkers for this type of cancer by validating gene-miRNA interactions and constructing bipartite networks. These miRNAs were not reported in the previous studies^31^.

Furthermore, another study used a similar method for discovering diagnostic biomarkers of the stratification of Breast Cancer molecular subtypes. It reported two or three miRNAs for each subtype and their target genes, which were significant and were highlighted in basic mechanisms of this cancer^32^. Moreover, there is a study that applied the WGCNA to find the key genes in Alzheimer’s disease and introduced them as potential targets in the therapy for this disease^33^. Another study in this field did not specifically deal with Alzheimer’s disease and concentrated on the aging of the brain. WGCNA was used in this study to identify the significant modules and effective biomarkers of the aging human brain^34^.

Many studies have been carried out in this field, and numerous proposed Alzheimer’s biomarkers have been introduced. However, the disorder remains incurable. This issue stems from the fact that its critical biological pathways, along with the involved functional genes, have not been fully discovered. For example, a recent research study applied WGCNA by focusing on gene targets and their pathways to investigate Alzheimer’s disease. However, it did not examine miRNAs^33^.

Therefore, our study broadens the scope of the previous studies and explores the relations between genes and their target miRNAs by constructing bipartite networks.

In this study, the exploration of the variations of genes expression between different steps by extracting related modules helps to find important interactions. Moreover, the examination of the related pathways helps us to develop a proper understanding of the development of Alzheimer’s disease. In the “Results” section, dataset details and the outcomes of the relevant experiments are illustrated and explained using the appropriate chart and tables. The “Discussion” section supports the applicability of the introduced method based on medical and clinical evidence. The proposed methods of our study are introduced in the “Methods” section, which is the last part of this article.

## Results

First, the details of the used database are presented, and the adopted methodological approaches are discussed step by step.

### Dataset and preprocessing

The dataset of this study was downloaded from the National Center for Biotechnology Information Gene expression Omnibus (GEO) using GSE63063 accession number. The platform of the chip analyzer was GPL6947.

First, before the preprocessing of the dataset, the non-gene transcripts were eliminated from the original file. Second, the remaining data were statically tested and preprocessed using “Limma” R package from the Bioconductor project, which was conducted in the RStudio ver.1.1.423 programming environment. Third, Benjamini & Hochberg’s false discovery rate method was applied to calculate the adjusted *p*-values. The genes were attributed to their related IDs, and the duplicated or ID-less genes were excluded. After sorting the genes according to their adjusted *p*-value, the significant genes with adjusted *p*-value < 0.01 were selected for further analyses, and the remaining ones were omitted. After preprocessing and removing outliers’ data, we determined 6,179 genes which were utilized as the gene list. This list enabled us to construct the network and perform further analysis.

**Figure 1:**
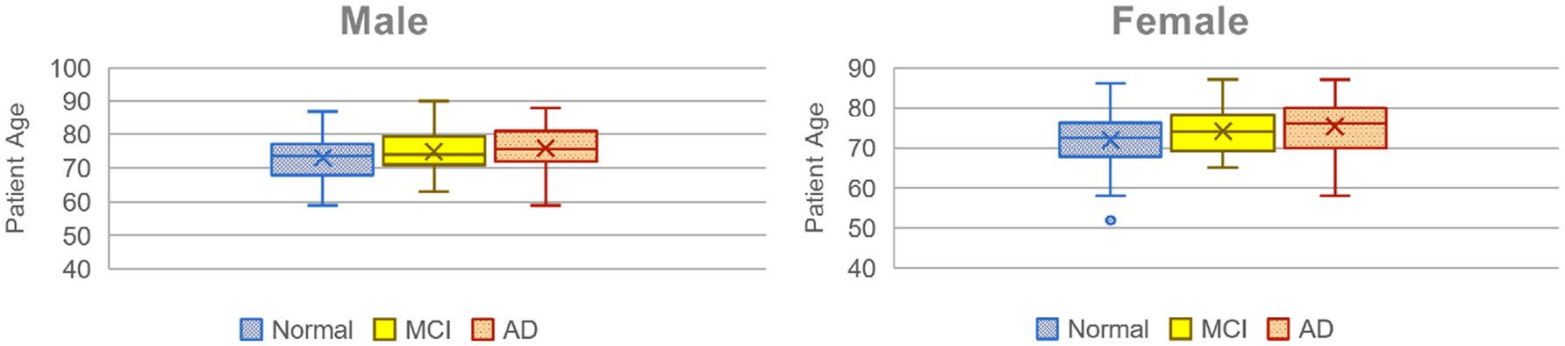
Dispersion of data samples according to their age and gender (*Normal* Healthy samples, *MCI* Mild Cognitive Impairment, *AD* Alzheimer’s Disease).

Moreover, our database was narrowed down to 104 normal samples, including 80 samples of mild cognitive impairment, and 145 samples of Alzheimer’s disease cases. The samples which were related to three stages of the disease were summed up in 329 samples, including 200 female and 129 male patients. The boxplots of Fig. 1 show the range and dispersion of samples according to age and gender at every three stages. Moreover, Table 1 shows the number of samples at each stage based on gender.

**Table 1:** Number of data samples according to their gender.

Gender	Alzheimer	Mild cognitive impairment	Normal
Male	46	41	42
Female	99	39	62
Total	145	80	104

### Weighted gene co-expression network analysis (WGCNA)

**Figure 2:**
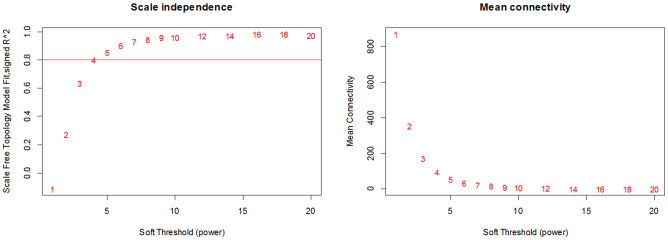
Scale independence measure is shown by R^2^ and the mean connectivity value to determine the optimal value of the soft threshold, which ensures a scale-free network and retains important correlations.

To construct the co-expression network, 6,179 genes from the 329 samples at three different stages were included. Figure 2 illustrates scale dependency by R^2^ and the mean connectivity, along with different values of the soft threshold. Among the powers which ranged from 1 to 20, the value of 4 was selected for β to gain the scale independency of the network at Normal stage, where the scale-free index R^2^ was 0.8. Consequently, the soft threshold values for the MCI stage and AD stage were set to 4 and 6 respectively (Supplementary Fig. S1 and S2).

Next, the adjacency matrix of the expression data was generated. Based on this matrix, we calculated our topological overlap matrix (TOM). The modules were selected using a tree-cut algorithm. Moreover, 4 and 20 values were used as *deepSplit* and *minimal module size* parameters respectively by examining different parameters. The extracted modules were merged in the following step and were labeled with colors. The threshold of 0.14 was chosen to merge the modules. The merged modules of all stages were presented in the Supplementary file (Supplementary Figs. S3–S5). The preservation measure, which is indicated in the Z_summary index, was used to select the effective modules. The modules were strongly preserved when the Z_summary value was equal to or larger than 10. The values which were between 2 and 10, were regarded to be moderately preserved. Finally, the values, which were equal to or smaller than 2, indicated the lack of preservation. The modules, whose Z_summary values were larger than 10, were strongly preserved and did not give us any information. Therefore, we did not use them.

**Figure 3:**
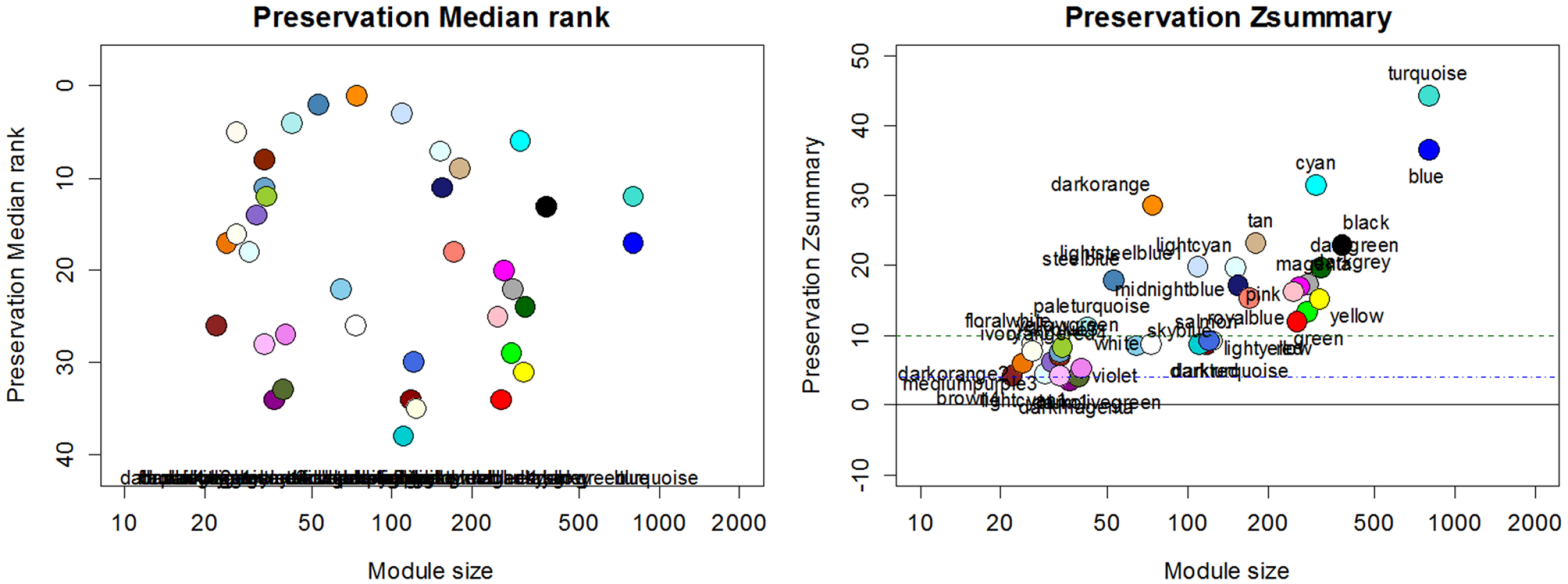
Modules of the Normal stage against the MCI expression data according to their Zsummary (The charts including Supplementary Figs. S6 and S7, which are related to the other two stages are available in the Supplementary file).

However, according to the obtained values, there were not any modules with z_summary values smaller than 2. Consequently, we selected 3, 5, and 3 as thresholds for Normal-MCI, MCI-AD, and Normal-AD module groups respectively. After choosing the thresholds, the Z_summary values of the selected modules were the values that ranged from 3.45 to 4.56 for Normal-MCI modules. Moreover, they ranged from 5.25 to 6.91 for MCI-AD modules and ranged from 3.41 to 5.33 for Normal-AD modules. To gain proper Zsummary values, we examined the deep-split parameter and the types of networks using separate execution procedures. Finally, the signed-hybrid network was set as the type of networks, and the value of deep-split was set to 4. Figure 3 illustrates the preservation of median rank and preservation of Z_summary along with the module size.

Five modules of the normal stage were selected in comparison with the MCI expression data (Normal-MCI modules). Zsummary values of these five modules were smaller than 4.5. Six modules of the MCI stage were chosen with Zsummary values smaller than 6.9 compared with the AD expression data (MCI-AD modules). Furthermore, six modules of the normal stage were selected compared with the AD expression data (Normal-AD modules) that had Zsummary values smaller than 5.3. The selected modules are illustrated by their attributes in Table 2.

**Table 2:** Extracted modules and their properties at three stages.

	Number of modules	Value of zsummary	Module color	Number of genes in module
**Normal**				
1	3	4.121447	Brown4	302
2	7	3.456988	Darkmagneta	36
3	8	4.066048	Darkolivegreen	39
4	19	4.568499	Lightcyan1	29
5	28	4,190,195	Plum1	33
**MCI**				
1	7	5.254516	Darkmagneta	26
2	8	6.120115	Darkolivegreen	27
3	20	6.261032	Paleturquoise	31
4	26	6.434162	Sienna3	26
5	28	6.918607	Steelblue	36
6	29	5.797834	Violet	28
**Alzheimer**				
1	3	4.455773	Brown4	22
2	7	3.410899	Darkmagneta	36
3	8	4.958944	Darkolivegreen	39
4	19	4.924977	Lightcyan1	29
5	28	4.263592	Plum1	33
6	37	5.335066	Violet	40

### Gene-miRNA bipartite network

**Figure 4:**
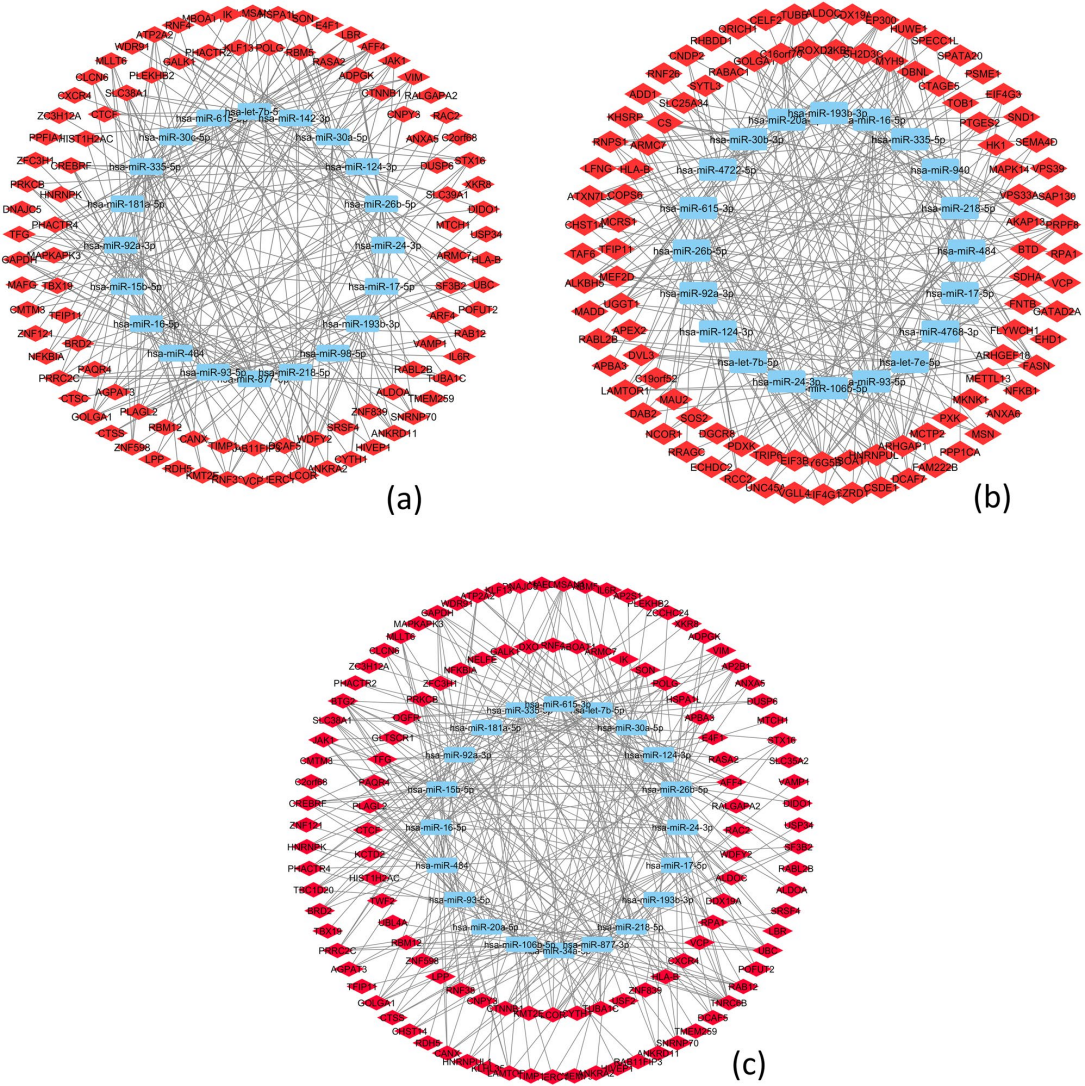
Bipartite gene-miRNA subnetworks for all of the three stages. Each subnetwork includes 20 miRNAs. (**a**) Normal-MCI subnetwork. (**b**) MCI-AD subnetwork and (**c**) Normal-AD subnetwork.

This part aims to analyze the relations among the obtained genes and their related miRNAs by which they are regulated. However, after constructing three bipartite networks, hub miRNAs with the highest connectivity degree were selected to reduce the complexity and to focus on the important connections. In this section, 20 miRNAs and their connections were selected. Therefore, the genes were also filtered by the ones that were at the end of this connection. In the obtained subnetworks, which are shown in Fig. 4, there were 116 genes of the Normal-MCI subnetwork, 131 genes of the MCI-AD subnetwork, and 145 genes of Normal-AD subnetwork.

## Enrichment analysis

### Enrichment analysis of genes

To extract the important pathways using the DAVID database, the pathways with normal *p-*value < 0.05 were selected as significant on the studied gene list. The *p-value* of the most significant pathway in our experiment was equal to 0.0028 and involved six genes. This pathway is called *Spliceosome* and is found in Normal-MCI subnetwork. In MCI-AD subnetwork, the *Herpes simplex infection* was our substantial pathway. Its *p-*value was equal to 0.0063, and it included seven significant genes. Similar to the first group, in the third subnetwork, called Normal-AD, the important pathway was *Spliceosome.* Its *p-*value was equal to 0.005 and involved six genes. The tables which were extracted for biological pathways are available in the Supplementary File (Supplementary Tables S1–S3).

Gene ontology analysis of these three subnetworks indicated that in the Normal-MCI subnetwork, the *regulation of the gene metabolic process* (*p-*value = 2.18e−04), which involved seven genes, was the most important process. *Vesicle-mediated transport* (*p-*value = 4.87e−04) with 22 genes, *apoptotic process* (*p-*value = 6.33e−04) with 24 genes, and *regulation of RNA splicing* (*p-*value = 6.89e−04) with 6 genes were the following important processes respectively. In the second subnetwork, called MCI-AD, *posttranscriptional regulation of gene expression* (*p-*value = 3.01e−05) with 14 genes, *regulation of translation* (*p-*value = 1.65e−04) with 11 genes, *regulation of cellular amide metabolic process* (*p-*value = 3.29e−04) with 11 genes, and *regulation of cellular protein metabolic process* (*p-*value = 7.70e−04) with 31 genes were the most important processes respectively. In the third subnetwork, called Normal-AD, *regulation of gene metabolic process* (*p-*value = 1.04e−06) with 10 genes, *gene metabolic process* (*p-*value = 7.60e−05) with 17 genes, *viral process* (*p-*value = 1.96e−04) with 20 genes, *multi-organism cellular process* (*p-*value = 2.15e−04) with 20 genes, *vesicle-mediated transport* (*p-*value = 2.28e−04) with 26 genes, *interspecies interaction between organisms* (*p-*value = 2.96e−04) with 20 genes, *symbiosis*, *encompassing mutualism through parasitism* (*p-*value = 2.96e−04) with 20 genes, and *positive regulation of gene metabolic process* (*p-*value = 6.85e−04) with 5 genes were the most significant processes respectively. All of the biological processes by related genes are available in Supplementary File (Supplementary Tables S4–S6).

### Enrichment analysis of miRNAs

At this stage, the hub miRNAs were evaluated using TAM tool^35^, and the type of *p*-value was normal. miRNAs found in Normal-MCI subnetwork were almost from the miR-15 and miR-17 family, and their *p-*values were 0.0097 and 0.0032, respectively. The most important functions in this subnetwork were *Apoptosis*, *Cell cycle-related*, *HIV latency*, *Hormones regulation*, *Human embryonic stem cell (hESC) regulation*, *cell proliferation,* and *immune system*. The second subnetwork was almost from the miR-17 family. Its *p-*value was equal to 1.54e−06. The important functions related to this subnetwork were *Angiogenesis*, *HIV latency*, *Hormones regulation*, *Human embryonic stem cell (hESC) regulation*, *cell proliferation,* and *immune system*. Finally, the third subnetwork was almost from the miR-15 family and miR-17 family. Its *p-*values were equal to 0.0097 and 0.0002, respectively. The important functions of this subnetwork were *Apoptosis*, *HIV latency*, *Hormones regulation*, *Human embryonic stem cell (hESC) regulation*, *cell proliferation,* and *immune system*. The related tables are available in Supplementary file (Supplementary Tables S7–S12).

### Detailed investigation over obtained biomarkers

First, the genes, which were regulated by the obtained miRNAs, were investigated. The first group involved nine genes that were obtained as a result of the intersection of three module groups. These genes were *GOLGA1*, *HLA-B*, *MBOAT1*, *RABL2B*, *ARMC7*, *IL10RB*, *STX5*, *TFIP11,* and *VCP*. The first one, *GoLGA1*, was introduced as one of the age-regulated genes^36^. The other gene, *HLA-B,* had high sensitivity and high specificity measurement values and was regarded to be a signal that showed the patients who suffered from hypersensitivity syndrome (HSS)^37^. One study compared AD and normal samples and revealed a significant difference in their HLA-B frequency^38^. The next gene, *RABL2B*, was introduced as a gene that is involved in a kind of neurological deficit called Phelan-McDermid Syndrome (PMS) ^39,40^.

*IL10RB* was obtained as one of the cell signaling molecules in the aging disease of the young population in 2014^41^. Another study used blood samples to determine early Alzheimer markers. It mentioned *IL10RB* as one of the best discriminators which distinguished between AD and normal samples^42^. A study in 2015 listed some previous studies that presented *STX5* as the protein which plays a role in Alzheimer and Parkinson diseases^43^. Another study examined protein–protein interaction networks and their impact on gene-network analysis using AD gene expression data. It introduced *TFIP11* as a significant hub gene^44^. The *VCP* gene is known to have a positive relationship with AD development risk based on the investigation of different types of dementia^45^. *VCP* mutations were investigated in another study that illustrated the vital role of these mutations in frontotemporal dementia^46^. Another study showed different genetic variants in genes like *VCP* which were associated with frontotemporal dementia and its related diseases^47^. According to another study, a mutation of *VCP* is related to Parkinson and Alzheimer diseases^48^.

The next group involved the genes which belonged to the overlap between MCI-AD module groups and CTL-AD module groups. There were eight genes in this group, including ALDOC, APBA3, CHST14, DDX19A, HNRNPUL1, KCTD2, LAMTOR1, and RPA1. The first one, ALDOC, was found to be related to Alzheimer’s disease in a study that investigated the proteomics in Alzheimer’s brain^49^. Another study, which discussed this gene, examined AD pathogenesis and its related key regulators^50^. The second gene, APBA3, and its interaction with beta-amyloid highlight the importance of the examination of its genomic structure^51^.

Similarly, another study investigated APBA3 as the gene which had a regulatory role in Alzheimer’s disease^52^. It has been shown that the third gene in this group, CHST14, and its relationship with impaired cognitive function affect the learning and memory abilities^53^. The next one, DDX19A, is among the AD-associated genes. This issue has been proved by imaging-wide association study (IWAS) and transcriptome-wide association study (TWAS)^54^. The next one, KCTD2, was found to be related to AD based on the results of a relevant study^55^. Another study, which focused on the genetic similarity between AD and Ischaemic Stroke (IS), found that KCTD2 was associated with both of these diseases^56^. The last gene in this group, PRA1, was mentioned in a study that compared the expression of nucleonic excision repair (NER) in AD according to brain tissues and blood. In both of these cases, RPA1 showed lower expression in AD samples in comparison with the healthy ones^57^. Two genes of this group, including HNRNPUL1 and LAMTOR1, have not been found in clinical research studies.

In this step, ten genes with larger degrees were selected from among the 107 genes which belonged to the overlap between CTL-MCI module groups and CTL-AD module groups to extend the exploration of the obtained results. These genes were PLAGL2, CREBRF, LCOR, ALDOA, LPP, KLF13, CANX, MRI1, STX16, and SLC38A1. One study showed that the variations of ALDOA were associated with Alzheimer’s disease^58^. Another study, which indicated the biomarkers of AD pathology, revealed that ALDOA was one of the obtained ones^59^. A different study showed that LPP was suppressed considerably in MCI samples in comparison with the healthy ones^60^. Another study named some of the genes that had regulation changes between healthy and AD samples and argued that LPP was one of them^61^. KLF13 was introduced in a neurodegenerative disease study as one of the key regulators in Alzheimer’s disease^62^. A similar study, which examined this disease between male and female samples, found that KLF13 existed in four male clinical traits in male patients^63^. The next one, CANX, was mentioned in a study as a target that had an important role in protein folding in AD cases^64^.

Moreover, it was mentioned in a comparative study on brain samples as an AD-related gene^65^. The other one, STX16, was among the genes which had expression changes in AD Frontal Cortex^66^ and was indicated in another study as the gene that showed common expression changes in AD samples^67^. Pathological survey on the role of mammalian target of rapamycin complex showed that SLC38A1 is one of the significant genes in neurodegenerative disease^68^. Finally, a study examined the potential functions of Amyloid β peptide, which has an important role in Alzheimer’s disease. It found that SLC38A1 was an effective gen^69^. Four of the genes in this group, including PLAGL2, CREBRF, LCOR, and MRI1, have not been found in clinical research studies.

In the following section, target miRNAs are investigated based on clinical research studies of Alzheimer’s disease. The first group involves thirteen miRNAs that were obtained based on the intersection of three module groups. These miRNAs were *miR-26b-5p, miR-335-5p, miR-92a-3p, miR-615-3p, miR-484, miR-16-5p, miR-17-5p, miR-218-5p, miR-24-3p, miR-124-3p, miR-93-5p, miR193-3p,* and *miR-20a-5p*.

The first miRNA was *mir-26b-5p*. According to the studies published in 2013 and 2018, the identification of the key miRNAs, which are associated with AD and *mir-26b-5p*, is also reported as one of the down-regulated key miRNAs^70, 71^. In another study, the authors reported the existence of a relationship between *mir-26b-5p* and Alzheimer’s disease and argued that this miRNA was upregulated in Alzheimer’s disease^72^. The *miR-26b-5p* was introduced as one of the signals which helped to distinguish sporadic behavioral variant of frontotemporal dementia from Alzheimer and healthy cases^73^. In two other studies, the researchers used *miR-26b-5p* as a previously known miRNA in brain diseases, especially Alzheimer’s disease^74,75^. In another study, which was published in 2017, *miR-26b-5p* was one of the significant miRNAs because of the dysregulation that it caused between AD and normal samples^76^. In two other studies, AD samples and normal samples were compared. These studies found that *miR-26b-5p* was one of the significant regulators for the identified differentially expressed genes^77,78^.

The second miRNA was *miR-335-5p.* This miRNA was reported as one of the miRNAs which were related to Alzheimer’s disease and represented the classifiers of Parkinson’s disease using dementia and Alzheimer’ samples^79^. There is also a study that represented *mir-335-5p* as an upregulated biomarker of AD^80^. The same miRNA was introduced as an upregulated biomarker in a different study^81^. Another study, which utilized neuroimages and investigated the concordance of miRNA biomarkers related to AD, identified *mir-335-5p* as an upregulated miRNA^82^. There is another study that used Low-Frequency Pulsed Electromagnetic Field (LF-PEMF) and found that *miR-335-5p* was a target miRNA and had a role in biological pathways of the Alzheimer’s disease^83^. Huynh, R.A., et al. investigated biomarkers of Alzheimer’s diseases in the genome, blood and cerebrospinal fluid and compared the AD samples with the normal ones. They observed that the gene expression level of *miR-335-5p* increased in the normal samples^84^.

The third miRNA, *miR-92a-3p*, was indicated as one of the regulator molecules which influenced transcriptional changes in Alzheimer’s disease^85^. In another study, *mir-92a-3p* was identified as one of the miRNAs that showed significant upregulation^86^. Similarly, in another study, the researchers mentioned that *mir-92a-3p* was a downregulated miRNA in serum samples of Alzheimer’s disease patients in comparison with the MCI patients’ serum samples^87^. In a different study, which measured miRNAs in the cerebrospinal fluid (CSF) and the blood of AD and MCI patients, *mir-92a-3p* was detected as the most frequent miRNAs in dementia patients^88^.

The fourth miRNA was *miR-615-3p.* In a study by Hoss, A.G, et al., this miRNA was detected as a significant signal of Huntington disease, which is a progressive brain disorder^89^. Likewise, a study by Hoss, A.G, et al. confirmed that *mir-615-3p* was a differentially expressed miRNA in Huntington disease^90^. Finally, Karnati, H.H., et al., reviewed the key causes of neurodegenerative disorders and epilepsy and emphasized the role of this miRNA in Huntington disease^91^.

The fifth miRNA was *miR-484.* In a study, it was determined to be an important miRNA which differed considerably between healthy individuals and MS^92^. Similarly, one of the previously-mentioned studies indicated that *miR-484* was an Alzheimer related miRNA^86^.

The sixth miRNA was *miR-16-5p*. It functioned as a deregulator in brain tissues based on a study that focused on Late-onset Alzheimer’s disease (LOAD)^93^. Another study investigated the effect of curcumin on Alzheimer’s disease and its neuroprotective role in this disease. The changes of the related miRNAs were assessed in this study. The study showed that *miR-16-5p* had a relationship with Alzheimer’s disease^94^. A different study investigated Frontotemporal Dementia (FTD). It used some circulating miRNAs and showed that *miR-16-5p* underwent significant changes from healthy individuals to FTD patients^95^. This miRNA was also recognized in Young-onset Alzheimer Disease (YOAD), which is recognized by clinical diagnosis before the age of 65^96^.

The next one, called *miR-17-5p*, has also been mentioned in many research studies that are related to Alzheimer’s disease. *Mir-17-5p* was considered as an important miRNA in recognition of FTD^95^. Study on the overlapping molecules of cancer and neurodegeneration showed that *miR-17-5p* and *miR-18d* are two gene regulators in neurotransmission^97^. The *miR-17-5p* was also determined as an AD-related miRNA^98^. Moreover, *miR-17-5p* was found to play an effective role in the production of the amyloid precursor protein (APP) and neuronal apoptosis which are two Alzheimer-related proteins^99^. A different study investigated *miR-17-5p* and its intersectional role in aging diseases and cancer^100^.

The next examined miRNA was *miR-218-5p*. As we examined clinical research studies, we found a study on the important miRNAs. The researchers of this study compared samples of MDD (Major Depressive Disorder), MCI, and AD patients. They argued that *miR-218-5p* was one of the ten top miRNAs whose expression differed conspicuously from MDD patients to MCI patients^101^.

One study used plasma exosomal miRNAs to find the effective miRNAs of Alzheimer. The researchers of this study argued that *miR-24-3p* was one of these miRNAs^102^. Another study that highlighted the diagnostic role of miRNAs in AD showed that *miR-24-3p* was an important signal in samples of cerebrospinal fluid (CSF) assays^77^. A different study expressed that *miR-24-3p* showed a considerable negative correlation between the expression levels in serum and CSF of the normal samples^73^. Another study examined the effects of the human microRNAome on modulating cellular prion protein (PrP^C^). The results of this study showed that *miR-124-3p* was an indirect regulator of PrP^C^^72^. An attractive study concluded that the regulation of *miR-124-3p* prevented the abnormal hyperphosphorylation of Tau protein^103^. In another study, the researchers found that *mir-124-3p* was among the other effective miRNAs and locus coeruleus (LC) was the most affected region that must be considered in future studies for further investigation^104^.

The *mir-93-5p* was the next miRNA which was found using serum data of AD and normal samples. A study showed that *miR-93-5p* had more changes in AD samples^105^. Another study revealed that *miR-93-5p* was one of the effective miRNAs in the case of MDD patients^106^.

The *miR-193b-3p* was identified in a study by comparing the AD samples with the normal ones. This study examined a decrease in the value of *miR-193b*^107^. The *miR-20a-5p* has also been examined in several articles using the network-based method and has been regarded as a regulator in AD samples^85^.

The second group had only one member which was miR-106b-5p. It belonged to the overlap between MCI-AD module groups and CTL-AD module groups. The examination of this miRNA shows that many studies have named it as a miRNA which is related to Alzheimer’s disease. There is a study that showed the radiation-induced changes of miRNA-106b-5p in the blood was involved in the development of Alzheimer’s diseas^108^. Another study proposed that miR-106b-5p was an upregulated miRNA in Alzheimer’s disease. This claim was validated using the qRT-PCR analysis^81^. Another study named this miRNA as a blood-based miRNA which was related to AD in more than 34 studies^109^.

The third group was miR-98-5p and was observed exclusively in CTL-MCI module group. This issue has been mentioned in some studies. For example, it was proposed as a novel therapeutic target for Alzheimer’s disease because of its crucial role in the accumulation of Aβ^110^. It has been shown that the expression levels of this miRNA differ considerably between normal samples and Alzheimer’s disease samples^111^.

The fourth group included 6 miRNAs which were observed exclusively in MCI-AD module groups. They were investigated one by one similar to the previous studies. Five miRNAs in this group have not been found in clinical research studies. They include miR-4722-5p, miR-4768-3p, miR-1827, miR-940, and miR-30b-3p. miR-106a-5p was introduced in a study that considered it as an effective miRNA in AD and used it to examine the effect of Huperzine-A on β-Amyloid peptide accumulation to determine the relationship between brain damage and neuro-muscular system deficiency^112^. A similar study used this miRNA as an effective miRNA in Alzheimer’s disease to express the Folic acid deficiency in amyloid-β accumulation^113^. Finally, a study showed that miR-106a-5p was an important biomarker of Alzheimer’s disease and argued that it was a predictor variable in AD^114^.

Likewise, the fifth group had 6 miRNAs which belonged to the intersection of CTL-MCI and CTL-AD module groups. The miRNA group members were miR-877-3p, miR-30a-5p, miR-30c-5p, miR-181a-5p, miR-142-3p and miR-15b-5p. The first one, miR-877-3p, was indicated as a miRNA which was effective in young-onset AD^96^. The second one, miR-30a-5p, was indicated as the miRNA which had a considerable and high expression in affected samples which were related to the early-onset familial Alzheimer’s disease^115^. Another study showed the effectiveness of miR-30a-5p for the same disease in the opposite direction brain-derived neurotrophic factor^116^. The next miRNA, mir-30c-5p, was introduced as a differentially expressed miRNA between normal and Alzheimer’s disease samples in two separate studies^81,117^. It was shown that the next miRNA, miR-181a-5p, was expressed at different levels in AD in comparison with the normal samples^118^. In another study, mir-181a-5p was shown to be an effective miRNA in Alzheimer’s disease using SNAP-25 vesicular protein^119^. The fifth one was mir-142-3p. It was introduced as one of the miRNAs which had considerably different levels in AD samples and normal samples^120^. A different study showed that the expression level of two target genes caused a reduction in the risk of AD by reducing the expression level of mir-142-3p^121^. The last one was mir-15b-5p. It was examined using one of the Alzheimer’s disease cell models named swAPP695-HEK293 and revealed the upregulation in the expression of mir-15b-5p^94^.

## Discussion

In this article, co-expression network analyses were performed for three stages of Alzheimer’s disease based on gene expressions. The experiments were performed on over 145 samples of Alzheimer stage, 80 samples of the MCI stage, and 104 samples which were at the healthy stage.

**Figure 5:**
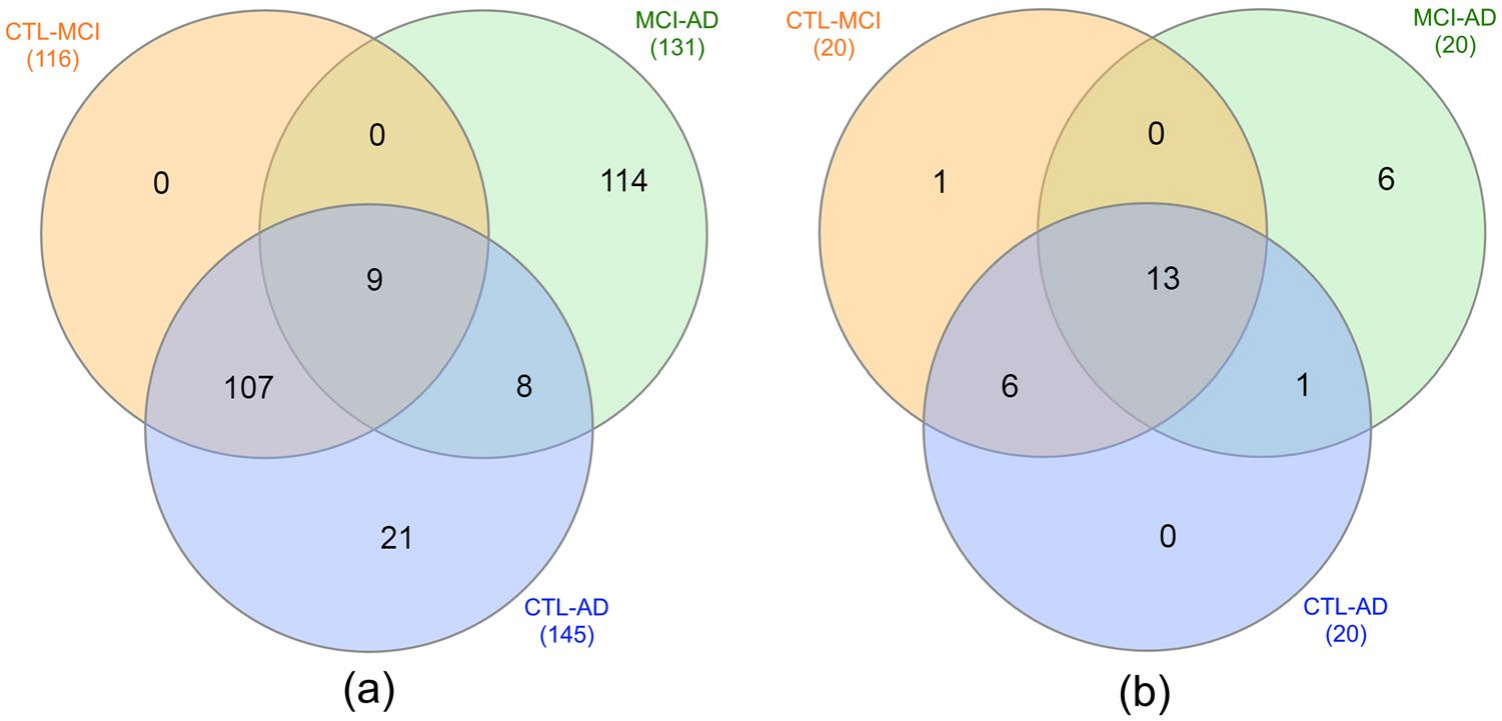
Venn diagram related to the extracted genes and miRNAs. (**a**) Venn diagram of genes (**b**) Venn diagram of miRNAs.

There were 6,179 genes in the total integrated dataset, which were generated by the genes with adjusted *p*-values smaller than 0.01. After network reconstruction, the modules were specified and merged to gain an optimal structure. Next, the target miRNAs that were related to the selected genes were extracted, and the bipartite networks were constructed for each stage. Subnetworks in the previous steps were constructed by selecting the hub miRNAs, which had pivotal roles in the regulation of the genes to reach optimal results. Then, the lists of extracted genes and miRNAs for each of the subnetworks were used to draw the Venn diagram and to indicate the intersections of three subnetworks. The related diagrams of genes and miRNAs are presented in Fig. 5. The list of the genes and miRNAs of these modules are shown separately by their different intersections and are listed in the Supplementary file as Supplementary Tables S13 and S14.

The listed genes and miRNAs in the above-mentioned tables were the proposed biomarkers of Alzheimer’s disease and were obtained using our proposed method. Therefore, we investigated the previous research studies and medical experiments as well as clinical studies in the field of Alzheimer’s disease.

The examination of the results of the clinical and experimental studies and the investigation of the recently published authentic articles show that almost all of our discovered genes and miRNAs have been reported in different studies of Alzheimer’s disease and neurological diseases. Most of them can be found in the latest articles. However, some of them have not been reported yet. Therefore, it can be claimed that the proposed biomarkers which were extracted using our methods can be real biomarkers that are related to Alzheimer’s disease and should be examined by experimental studies. Some of our proposed biomarkers have been reported in the aging disease that is related to Alzheimer’s disease. Eight of the discovered genes in this study, including *MBOAT1*, *ARMC7*, *HNRNPUL1*, *LAMTOR1, PLAGL2, CREBRF, LCOR,* and *MRI1,* have not been reported in the recent authentic Alzheimer related articles. RABL2B has been reported as a gene that is related to Phelan-McDermid Syndrome (PMS) and is not associated with Alzheimer. Therefore, these genes are the landmark finding of our study, and we propose them as the biomarkers of Alzheimer’s disease.

Moreover, we introduced Mir-615-3p as an Alzheimer-related miRNA which has been recognized as a biomarker of the Huntington disease. Furthermore, we dealt with five miRNAs, including miR-4722-5p, miR-4768-3p, miR-1827, miR-940, and miR-30b-3p, which have not been reported in the previous studies. Consequently, they can be considered as new proposed biomarkers which have to be examined by clinical experiments. Finally, we speculated that our gathered biomarkers, which were prepared in tables in the Supplementary file (Supplementary Tables S13 and S14), can be studied as potential biomarkers for the early detection of Alzheimer’s disease.

In summary, our proposed method aimed to conduct a prognostic study of Alzheimer’s disease and used the Gene co-expression Network analysis method based on the GEO database. To this end, significant modules obtained from co-expression networks were utilized to construct bipartite networks (gene-miRNA) for the three stages of the disease. Therefore, we worked with three types of samples. Each type of sample belonged to one of the *normal*, *mild cognitive impairment*, and *Alzheimer’s disease* stages. This study identified the hub genes. These genes have the highest connectivity degrees and are regarded to be the potential prognostic biomarkers for Alzheimer’s disease. The novel genes, including MBOAT1, ARMC7, RABL2B, HNRNPUL1, LAMTOR1, PLAGL2, CREBRF, LCOR, and MRI1 together with miRNAs comprising miR-615-3p, miR-4722-5p, miR-4768-3p, miR-1827, miR-940, and miR-30b-3p are introduced as Alzheimer-related proposed biomarkers and should be examined in the experimental studies as clinical studies. This study points out that its exploration needs further research to develop novel therapeutic approaches to drug design and drug discovery along with medical approaches to the treatment of Alzheimer’s disease.

## Methods

This part explains the methods which were used in this study step by step.

### Networks construction

To construct networks, we detected the outlier samples using an optimal version of hierarchical clustering, which uses distance and averaging methods to cluster the study samples. The results of the clustering approach showed that there were only two outliers at the AD stage. Moreover, they revealed that there were not any outliers at the other two stages. The WGCNA approach was utilized to perform an analysis of the gene expression data. The Co-expression networks were constructed for all of the genes at each of the stages separately. In the next step, mean connectivity and scale dependency measures were calculated to choose the proper soft power and to reconstruct the network. Lastly, soft threshold power was evaluated using network analysis functions to preserve more correlated genes based on scale-free topology.

### Module extraction

The dissimilarity matrix was obtained from TOM matrix to apply the module analysis algorithm. This matrix was used to perform a hierarchical clustering to recognize the potential modules. The modules were selected by using a tree-cut algorithm and experimenting with different values for *deepSplit* and *minimal module size* parameters. The extracted modules were merged and labeled with colors. To merge the modules, we extracted eigengenes of modules. After calculating the dissimilarity of the eigengenes, the clustering method was applied to eigengenes.

At this point, module preservation analysis was performed to identify the meaningful modules at different stages of the disease. Module preservation function specified the amount of changes in the modules against the network of the next stage by calculating Zsummary. A large change was observed in the modules when the value of Zsummary was small. This issue was in line with the aim of this study since we preferred to find the modules that underwent bigger changes.

### Bipartite gene-miRNA networks

In the next step, the genes of the Normal-MCI modules were merged and were experimentally validated. Moreover, the target miRNAs of these genes were extracted using the miRWalk2.0 database. The MCI-AD modules and Normal-AD modules underwent the same process. Three bipartite gene-miRNA networks were constructed by the genes and their target miRNAs by Cytoscape.3.7.0. The miRNAs with larger degrees had more connections with the selected genes and performed more regulatory roles in the network. Therefore, 20 miRNAs with the highest degree values were chosen together with their connections and were used to reconstruct the network.

### Functional enrichment analysis

The Annotation Visualization and Integrated Discovery (DAVID) database was used to study the biological mechanism and gene ontology of the selected genes^122,123^. The biological processes of the selected genes were listed, and the nodes (*p-*value < 0.01) were reported as important processes. Kyoto Encyclopedia Gene and Genomes (KEGG) database^124^ was used to perform the pathway enrichment analysis and the significant genes (*p-*value < 0.05) were selected.

## Supplementary information

Supplementary Information.

## Data Availability

The datasets which were processed in the present study can be provided by the corresponding author on reasonable request. The raw dataset is available on Information Gene expression Omnibus (GEO) with GSE63063 accession number ( https://www.ncbi.nlm.nih.gov/geo/query/acc.cgi?acc=GSE63063).
