# Supplementary material for: Gene biomarker discovery at different stages of Alzheimer using gene co-expression network approach
Source: Sci Rep. 2020 Jul 22;10:12210. doi: 10.1038/s41598-020-69249-8 (PMC7376049; doi:10.1038/s41598-020-69249-8)
Supplement: Supplementary file 1 — Supplementary Information. [file 41598_2020_69249_MOESM1_ESM.docx]

**Gene biomarker discovery at different stages of Alzheimer using Gene Co-Expression Network approach**

Negar Sadat Soleimani Zakeri^1^, Saeid Pashazadeh^2*^, Habib MotieGhader^3^

1, 2. Faculty of Electrical and Computer Engineering, University of Tabriz, Tabriz, Iran.

3. Department of Computer Engineering, Gowgan Educational Center, Tabriz Branch, Islamic Azad University, Tabriz, Iran.

* Corresponding author: Saeid Pashazadeh; contact info: [pashazadeh@tabrizu.ac.ir](mailto:pashazadeh@tabrizu.ac.ir); Work phone number: +984133393790; Work fax number: +984133300829


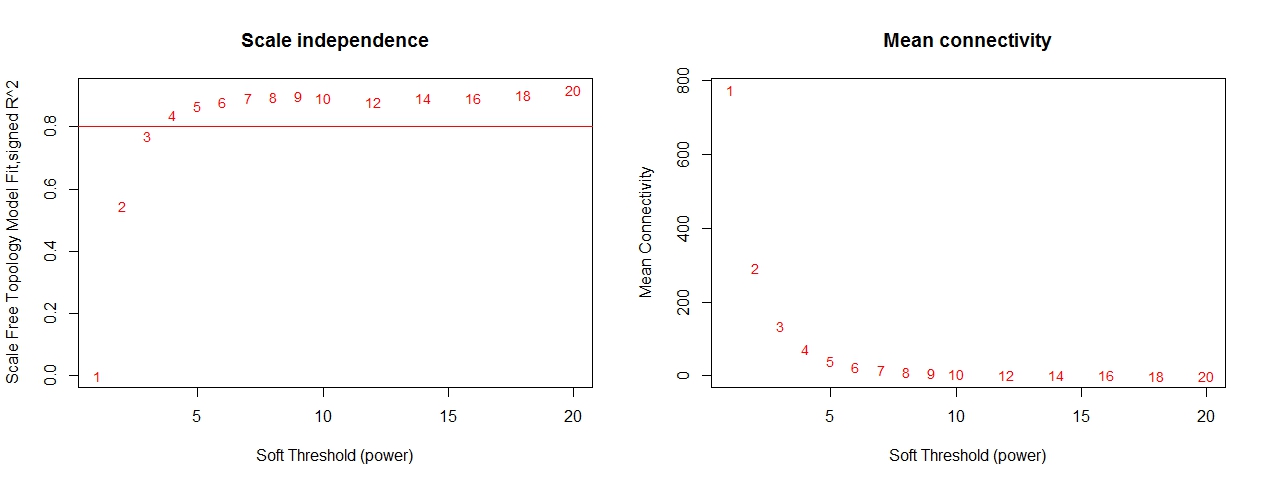


**Supplementary Figure S1.** Scale independence measure shown by R^2^ and mean connectivity value in the MCI stage.


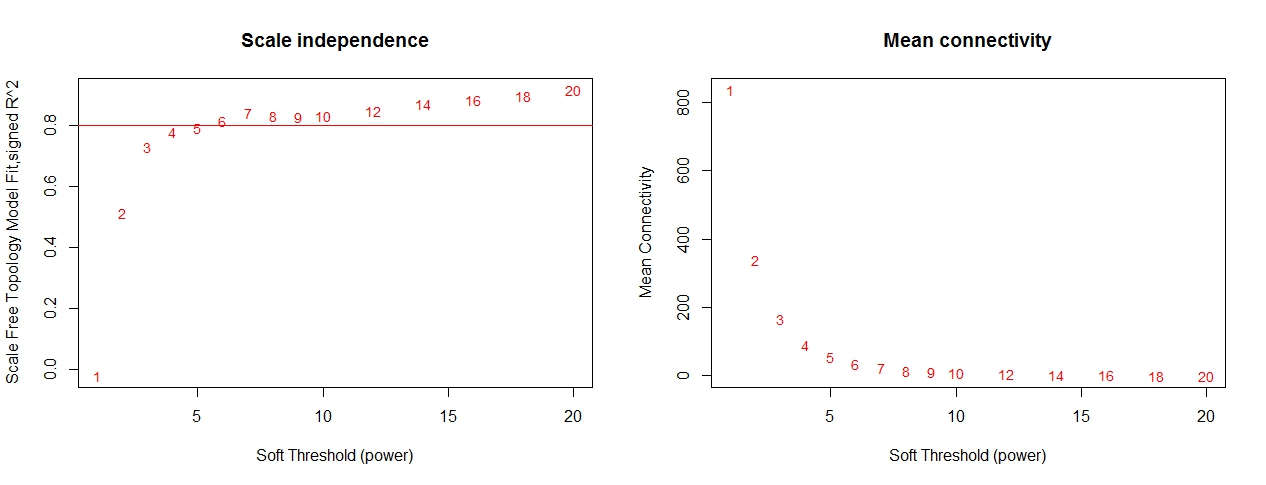


**Supplementary** **Figure S2.** Scale independence measure shown by R^2^ and mean connectivity value in AD Stage.


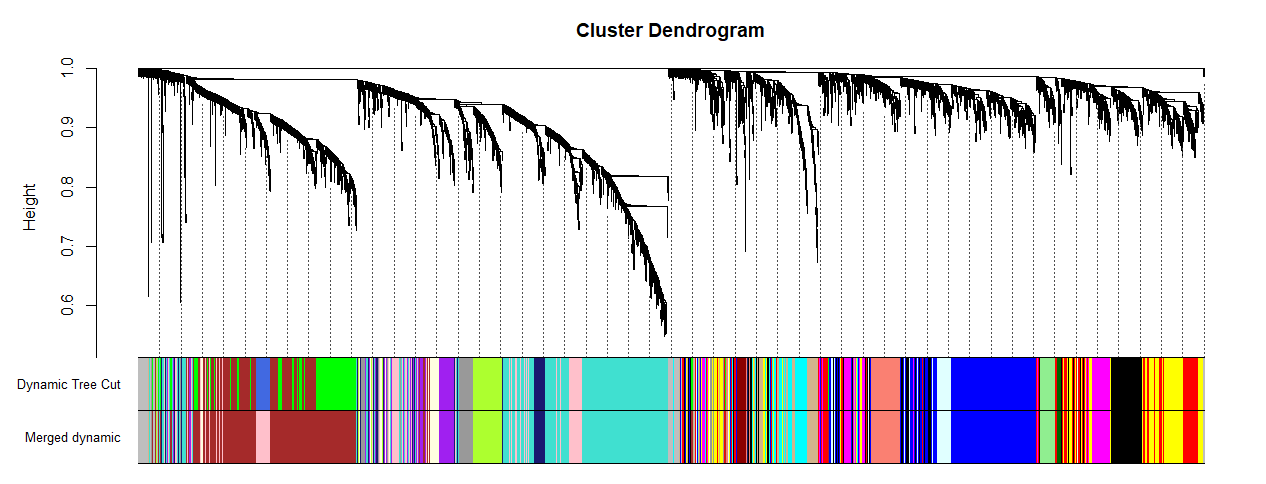

**Supplementary Figure S3.** Merging of extracted modules in Normal Stage.


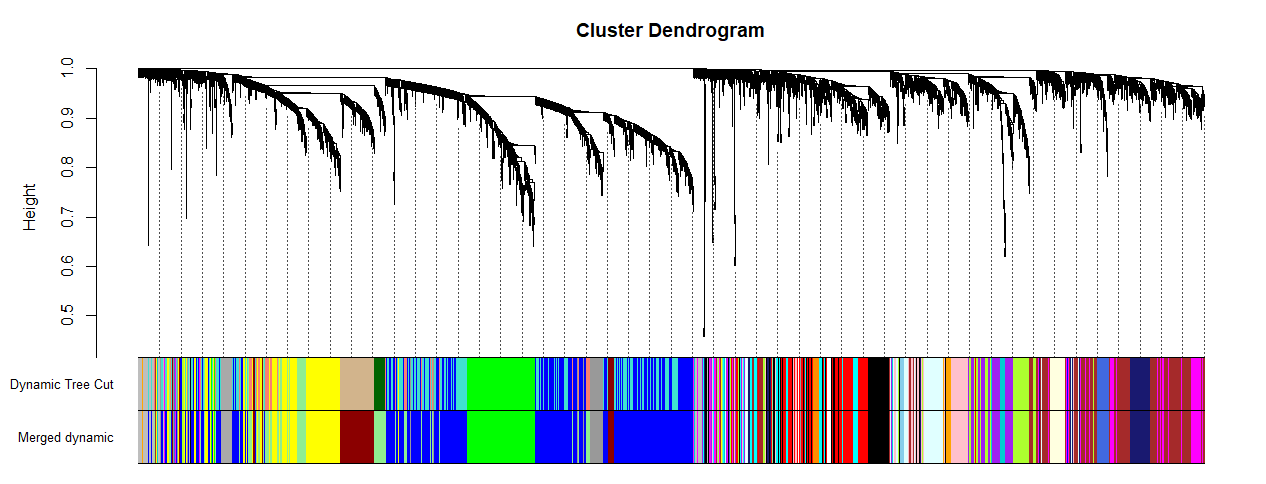


**Supplementary Figure S4.** Merging of extracted modules in MCI Stage.


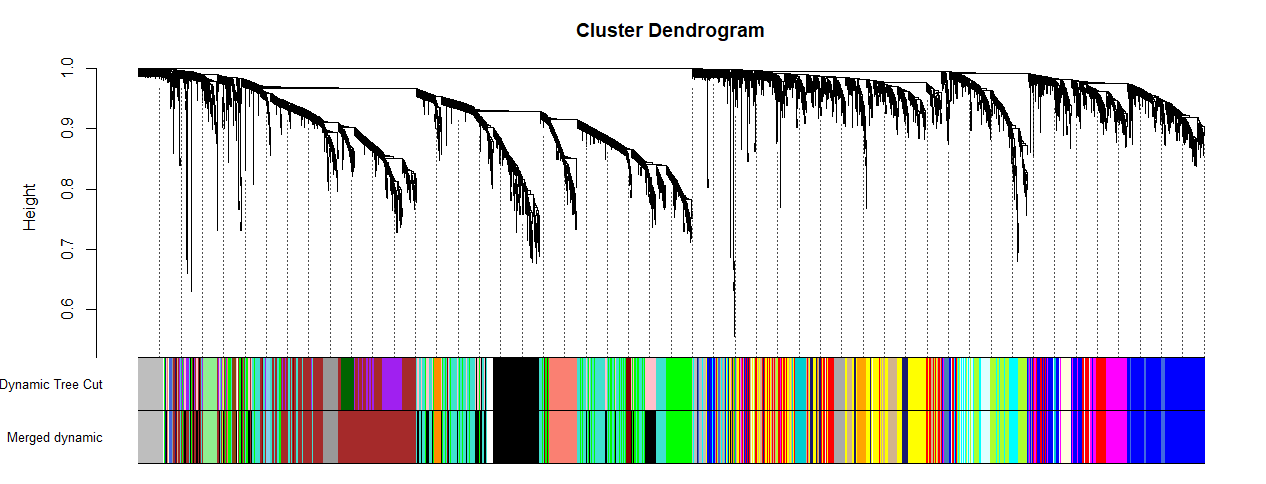


**Supplementary Figure S5.** Merging of extracted modules in AD Stage.


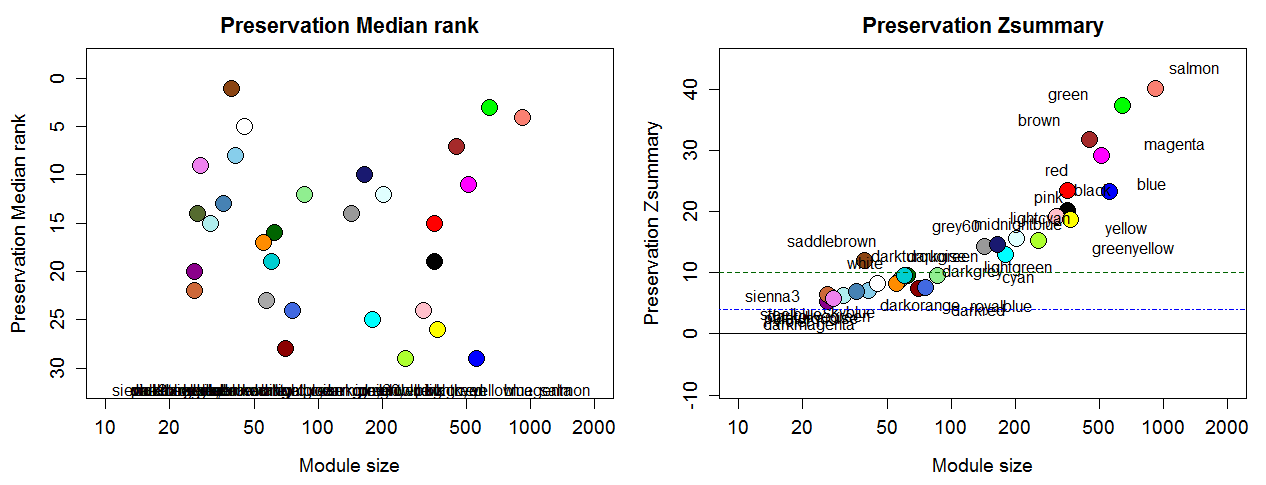


**Supplementary Figure S6.** Modules of the MCI stage against the AD expression data according to their Zsummary.


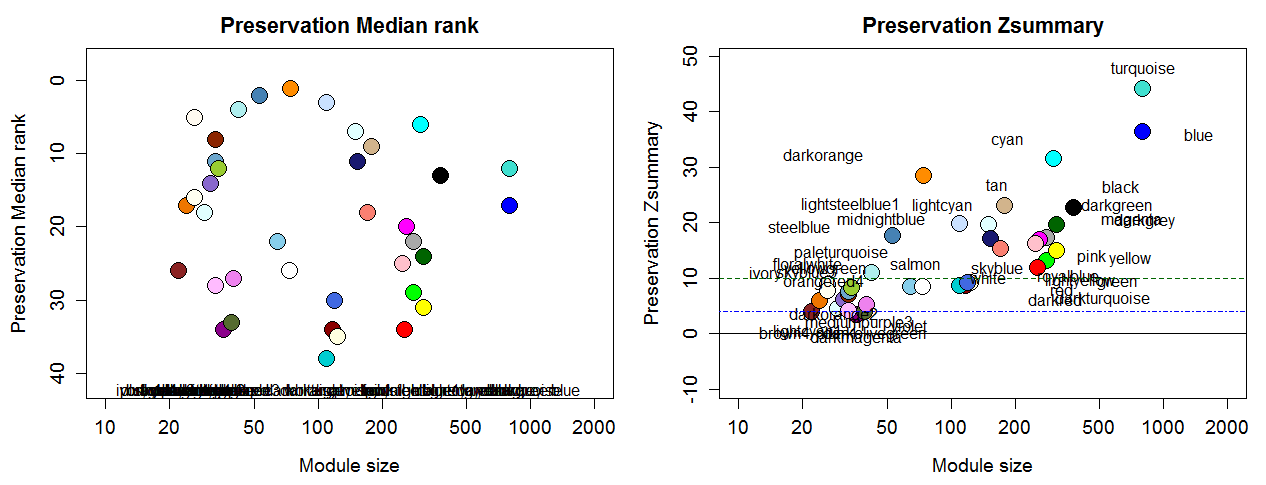


**Supplementary Figure S7.** Modules of Normal stage against the AD expression data according to their Zsummary.

| Term | Count | PValue | Genes |
| --- | --- | --- | --- |
| Spliceosome | 6 | 0.002885 | HSPA1L, SRSF4, HNRNPK, PCBP1, SNRNP70, SF3B2 |
| Antigen processing and presentation | 4 | 0.018466 | HSPA1L, HLA-B, CTSS, CANX |
| SNARE interactions in vesicular transport | 3 | 0.025993 | STX5, STX16, VAMP1 |
| HIF-1 signaling pathway | 4 | 0.033867 | IL6R, GAPDH, PRKCB, TIMP1 |
| MAPK signaling pathway | 6 | 0.038397 | HSPA1L, RAC2, MAPKAPK3, RASA2, PRKCB, DUSP6 |
| HTLV-I infection | 6 | 0.038954 | ZFP36, NFKBIA, JAK1, HLA-B, CANX, CTNNB1 |
| Herpes simplex infection | 5 | 0.045943 | SRSF4, HNRNPK, NFKBIA, JAK1, HLA-B |
| Toxoplasmosis | 4 | 0.047623 | HSPA1L, IL10RB, NFKBIA, JAK1 |

**Supplementary Table S1.** Biological pathways related to the mRNAs in Normal-MCI subnetwork KEEG_PATHWAY database.

| Term | Count | PValue | Genes |
| --- | --- | --- | --- |
| Herpes simplex infection | 7 | 0.006313 | PPP1CA, MCRS1, MYD88, EP300, TAF6, NFKB1, HLA-B |
| Insulin signaling pathway | 6 | 0.008365 | PPP1CA, SOS2, FASN, MKNK1, CALM3, HK1 |
| Legionellosis | 4 | 0.013032 | MYD88, VCP, NFKB1, TLR5 |
| Carbon metabolism | 5 | 0.019566 | SDHA, ACO1, ALDOC, CS, HK1 |
| Tuberculosis | 6 | 0.022517 | MYD88, EP300, IL10RB, MAPK14, CALM3, NFKB1 |
| Citrate cycle (TCA cycle) | 3 | 0.030461 | SDHA, ACO1, CS |
| Pertussis | 4 | 0.030998 | MYD88, MAPK14, CALM3, NFKB1 |
| Salmonella infection | 4 | 0.040078 | MYD88, MAPK14, NFKB1, TLR5 |
| Biosynthesis of antibiotics | 6 | 0.044015 | SDHA, FNTB, ACO1, ALDOC, CS, HK1 |
| Prostate cancer | 4 | 0.046365 | EP300, SOS2, CREB5, NFKB1 |

**Supplementary Table S2.** Biological pathways related to the mRNAs in MCI-AD subnetwork KEEG_PATHWAY database.

| Term | Count | PValue | Genes |
| --- | --- | --- | --- |
| Spliceosome | 6 | 0.00505455 | HSPA1L, SRSF4, HNRNPK, PCBP1, SNRNP70, SF3B2 |
| Endocytosis | 7 | 0.015347803 | HSPA1L, AP2B1, RAB11FIP3, CYTH1, CXCR4, AP2S1, HLA-B |
| Glycolysis / Gluconeogenesis | 4 | 0.018613712 | ALDOA, ADPGK, ALDOC, GAPDH |
| Antigen processing and presentation | 4 | 0.025905279 | HSPA1L, HLA-B, CTSS, CANX |
| SNARE interactions in vesicular transport | 3 | 0.032984831 | STX5, STX16, VAMP1 |
| HIF-1 signaling pathway | 4 | 0.046828715 | IL6R, GAPDH, PRKCB, TIMP1 |

**Supplementary Table S3.** Biological pathways related to the mRNAs in MCI-AD subnetwork in KEEG_PATHWAY database.

| Term | Count | PValue | Genes |
| --- | --- | --- | --- |
| GO:1903311~regulation of mRNA metabolic process | 7 | 2.18E-04 | ZFP36, SON, SRSF4, HNRNPK, RBM5, ZC3H12A, SNRNP70 |
| GO:0016192~vesicle-mediated transport | 22 | 4.87E-04 | ALDOA, STX5, PHACTR2, CYTH1, XKR8, MAPKAPK3, TFG, CANX, TIMP1, CTNNB1, RAB11FIP3, HNRNPK, RAC2, ATP2A2, VCP, CD93, STX16, ARF4, GOLGA1, CTSC, DNAJC5, VAMP1 |
| GO:0006915~apoptotic process | 24 | 6.33E-04 | ZFP36, XKR8, RBM5, NFKBIA, IL6R, ANXA5, DIDO1, PRKCB, TIMP1, CTNNB1, SON, NOD2, HNRNPK, VCP, CXCR4, MTCH1, ARF4, UBC, ZC3H12A, CTSC, DNAJC5, GAPDH, DUSP6, PLAGL2 |
| GO:0043484~regulation of RNA splicing | 6 | 6.89E-04 | SON, SRSF4, HNRNPK, AKAP17A, RBM5, SNRNP70 |
| GO:0048024~regulation of mRNA splicing, via spliceosome | 5 | 0.001070831 | SON, SRSF4, HNRNPK, RBM5, SNRNP70 |
| GO:0008219~cell death | 25 | 0.001300081 | RBM5, NFKBIA, TMEM259, DIDO1, CTNNB1, TIMP1, NOD2, HNRNPK, CXCR4, MTCH1, ZC3H12A, DNAJC5, GAPDH, PLAGL2, ZFP36, XKR8, IL6R, ANXA5, PRKCB, SON, VCP, ARF4, UBC, CTSC, DUSP6 |
| GO:0012501~programmed cell death | 24 | 0.001405766 | ZFP36, XKR8, RBM5, NFKBIA, IL6R, ANXA5, DIDO1, PRKCB, TIMP1, CTNNB1, SON, NOD2, HNRNPK, VCP, CXCR4, MTCH1, ARF4, UBC, ZC3H12A, CTSC, DNAJC5, GAPDH, DUSP6, PLAGL2 |
| GO:0034097~response to cytokine | 14 | 0.001902051 | ZFP36, CREBRF, MAPKAPK3, NFKBIA, IL6R, HLA-B, TIMP1, CXCR4, IL10RB, UBC, ZC3H12A, JAK1, SNRNP70, GAPDH |
| GO:0060255~regulation of macromolecule metabolic process | 53 | 0.002508958 | CREBRF, TAF1C, RBM5, CTCF, CTNNB1, NOD2, AKAP17A, CXCR4, PCBP1, RNF38, E4F1, ELMSAN1, TFIP11, ZFP36, STX5, ANKRA2, IL6R, PRKCB, UBC, CTSC, LCOR, TBX19, KMT2E, HIST1H2AC, VIM, MAPKAPK3, NFKBIA, TMEM259, TIMP1, HNRNPK, RAC2, MTCH1, ZC3H12A, SPIDR, SNRNP70, USP34, MLLT6, GAPDH, PLAGL2, MAFG, WDFY2, BRD2, KLF13, ZNF121, AFF4, SON, SRSF4, VCP, RNF4, ARF4, HIVEP1, POFUT2, DUSP6 |
| GO:0010604~positive regulation of macromolecule metabolic process | 31 | 0.003418643 | KMT2E, CREBRF, TAF1C, VIM, MAPKAPK3, NFKBIA, CTCF, TMEM259, CTNNB1, NOD2, HNRNPK, CXCR4, PCBP1, MTCH1, ZC3H12A, SPIDR, SNRNP70, PLAGL2, MAFG, ZFP36, STX5, WDFY2, KLF13, IL6R, RNF4, VCP, ARF4, UBC, CTSC, TBX19, DUSP6 |
| GO:0002221~pattern recognition receptor signaling pathway | 6 | 0.00364117 | NOD2, MAPKAPK3, CNPY3, UBC, NFKBIA, CTSS |
| GO:0008380~RNA splicing | 9 | 0.004290214 | TFIP11, SON, SRSF4, HNRNPK, AKAP17A, PCBP1, RBM5, SNRNP70, SF3B2 |
| GO:0043122~regulation of I-kappaB kinase/NF-kappaB signaling | 7 | 0.004379904 | NOD2, UBC, NFKBIA, ZC3H12A, TFG, CTNNB1, PRKCB |
| GO:0050684~regulation of mRNA processing | 5 | 0.004546198 | SON, SRSF4, HNRNPK, RBM5, SNRNP70 |
| GO:0051650~establishment of vesicle localization | 7 | 0.004747219 | STX5, ATP2A2, TFG, CTSC, DNAJC5, CANX, CTNNB1 |
| GO:0010033~response to organic substance | 30 | 0.005603358 | KMT2E, CREBRF, MAPKAPK3, NFKBIA, TMEM259, CTNNB1, TIMP1, HSPA1L, NOD2, CXCR4, IL10RB, ZC3H12A, SPIDR, SNRNP70, GAPDH, ZFP36, POLG, CTSS, IL6R, HLA-B, ANXA5, PRKCB, SRSF4, RNF4, VCP, ATP2A2, UBC, JAK1, CTSC, DUSP6 |
| GO:0009896~positive regulation of catabolic process | 8 | 0.005720979 | ZFP36, CREBRF, STX5, VCP, UBC, ZC3H12A, CTSC, TMEM259 |
| GO:0071345~cellular response to cytokine stimulus | 12 | 0.005721724 | ZFP36, CREBRF, CXCR4, IL10RB, UBC, NFKBIA, ZC3H12A, JAK1, SNRNP70, HLA-B, IL6R, GAPDH |
| GO:0030163~protein catabolic process | 13 | 0.005887963 | CREBRF, STX5, CTSS, TMEM259, TIMP1, CTNNB1, VCP, RNF4, RNF38, UBC, RAB12, CTSC, USP34 |
| GO:0051252~regulation of RNA metabolic process | 37 | 0.005888347 | KMT2E, CREBRF, HIST1H2AC, TAF1C, RBM5, NFKBIA, CTCF, CTNNB1, NOD2, AKAP17A, HNRNPK, PCBP1, RNF38, ZC3H12A, E4F1, SNRNP70, MLLT6, USP34, PLAGL2, ELMSAN1, TFIP11, ZFP36, MAFG, BRD2, ANKRA2, KLF13, ZNF121, AFF4, PRKCB, SON, SRSF4, RNF4, ARF4, UBC, HIVEP1, LCOR, TBX19 |
| GO:0051648~vesicle localization | 7 | 0.005985701 | STX5, ATP2A2, TFG, CTSC, DNAJC5, CANX, CTNNB1 |
| GO:0006357~regulation of transcription from RNA polymerase II promoter | 22 | 0.006125424 | ZFP36, MAFG, CREBRF, BRD2, ANKRA2, KLF13, NFKBIA, CTCF, PRKCB, CTNNB1, NOD2, HNRNPK, RNF4, PCBP1, ARF4, UBC, ZC3H12A, HIVEP1, E4F1, TBX19, ELMSAN1, PLAGL2 |
| GO:0007249~I-kappaB kinase/NF-kappaB signaling | 7 | 0.006329052 | NOD2, UBC, NFKBIA, ZC3H12A, TFG, CTNNB1, PRKCB |
| GO:0002376~immune system process | 27 | 0.006474911 | KMT2E, MAPKAPK3, NFKBIA, PRRC2C, CANX, CTNNB1, NOD2, AKAP17A, RAC2, CD93, IL10RB, CXCR4, ZC3H12A, GAPDH, ZFP36, IK, KLF13, CNPY3, CTSS, ANKHD1, IL6R, HLA-B, PRKCB, UBC, JAK1, CTSC, CMTM3 |
| GO:0065009~regulation of molecular function | 30 | 0.006875519 | CYTH1, MAPKAPK3, NFKBIA, CTCF, ADAP1, CTNNB1, TIMP1, NOD2, TAGAP, RAC2, CXCR4, MTCH1, ZC3H12A, SNRNP70, RASA2, ZFP36, TFIP11, PHACTR2, PHACTR4, CTSS, IL6R, ANXA5, HERC1, PRKCB, RALGAPA2, VCP, ARF4, UBC, JAK1, DUSP6 |
| GO:0002224~toll-like receptor signaling pathway | 5 | 0.007297319 | MAPKAPK3, CNPY3, UBC, NFKBIA, CTSS |
| GO:0080090~regulation of primary metabolic process | 51 | 0.007390309 | CREBRF, TAF1C, RBM5, CTCF, CTNNB1, NOD2, AKAP17A, CXCR4, PCBP1, RNF38, E4F1, ELMSAN1, TFIP11, ZFP36, STX5, ANKRA2, IL6R, PRKCB, UBC, CTSC, LCOR, TBX19, KMT2E, HIST1H2AC, MAPKAPK3, NFKBIA, TMEM259, TIMP1, HNRNPK, RAC2, MTCH1, ZC3H12A, SPIDR, SNRNP70, USP34, MLLT6, GAPDH, PLAGL2, MAFG, WDFY2, BRD2, KLF13, ZNF121, AFF4, SON, SRSF4, VCP, RNF4, ARF4, HIVEP1, DUSP6 |
| GO:0010941~regulation of cell death | 19 | 0.007498557 | ZFP36, RBM5, NFKBIA, TMEM259, ANXA5, TIMP1, CTNNB1, SON, NOD2, HNRNPK, VCP, MTCH1, ARF4, UBC, ZC3H12A, CTSC, DNAJC5, DUSP6, PLAGL2 |
| GO:0019219~regulation of nucleobase-containing compound metabolic process | 39 | 0.007872607 | KMT2E, CREBRF, HIST1H2AC, TAF1C, RBM5, NFKBIA, CTCF, CTNNB1, NOD2, HNRNPK, AKAP17A, PCBP1, RNF38, ZC3H12A, SPIDR, E4F1, SNRNP70, USP34, MLLT6, PLAGL2, ELMSAN1, TFIP11, ZFP36, MAFG, BRD2, ANKRA2, KLF13, ZNF121, AFF4, PRKCB, SON, SRSF4, RNF4, VCP, ARF4, UBC, HIVEP1, LCOR, TBX19 |
| GO:0042981~regulation of apoptotic process | 18 | 0.007909643 | ZFP36, RBM5, NFKBIA, ANXA5, TIMP1, CTNNB1, SON, NOD2, HNRNPK, VCP, MTCH1, ARF4, UBC, ZC3H12A, CTSC, DNAJC5, DUSP6, PLAGL2 |
| GO:0019362~pyridine nucleotide metabolic process | 5 | 0.008182138 | ALDOA, GALK1, VCP, ADPGK, GAPDH |
| GO:0046496~nicotinamide nucleotide metabolic process | 5 | 0.008182138 | ALDOA, GALK1, VCP, ADPGK, GAPDH |
| GO:0006096~glycolytic process | 4 | 0.008465108 | ALDOA, GALK1, ADPGK, GAPDH |
| GO:0045088~regulation of the innate immune response | 8 | 0.0086238 | NOD2, MAPKAPK3, CNPY3, UBC, NFKBIA, JAK1, HLA-B, CTSS |
| GO:0043067~regulation of programmed cell death | 18 | 0.008640476 | ZFP36, RBM5, NFKBIA, ANXA5, TIMP1, CTNNB1, SON, NOD2, HNRNPK, VCP, MTCH1, ARF4, UBC, ZC3H12A, CTSC, DNAJC5, DUSP6, PLAGL2 |
| GO:0006757~ATP generation from ADP | 4 | 0.00882733 | ALDOA, GALK1, ADPGK, GAPDH |
| GO:0016032~viral process | 14 | 0.009038956 | ZFP36, HNRNPK, CD93, VCP, CXCR4, IL10RB, PCBP1, VIM, UBC, NFKBIA, ZC3H12A, HLA-B, E4F1, SF3B2 |
| GO:0009893~positive regulation of metabolic process | 31 | 0.009042832 | KMT2E, CREBRF, TAF1C, VIM, MAPKAPK3, NFKBIA, CTCF, TMEM259, CTNNB1, NOD2, HNRNPK, CXCR4, PCBP1, MTCH1, ZC3H12A, SPIDR, SNRNP70, PLAGL2, MAFG, ZFP36, STX5, WDFY2, KLF13, IL6R, RNF4, VCP, ARF4, UBC, CTSC, TBX19, DUSP6 |
| GO:0044764~multi-organism cellular process | 14 | 0.009569467 | ZFP36, HNRNPK, CD93, VCP, CXCR4, IL10RB, PCBP1, VIM, UBC, NFKBIA, ZC3H12A, HLA-B, E4F1, SF3B2 |
| GO:0019222~regulation of metabolic process | 53 | 0.009790549 | CREBRF, TAF1C, RBM5, CTCF, CTNNB1, NOD2, AKAP17A, CXCR4, PCBP1, RNF38, E4F1, ELMSAN1, TFIP11, ZFP36, STX5, ANKRA2, IL6R, PRKCB, UBC, CTSC, LCOR, TBX19, KMT2E, HIST1H2AC, VIM, MAPKAPK3, NFKBIA, TMEM259, TIMP1, HNRNPK, RAC2, MTCH1, ZC3H12A, SPIDR, SNRNP70, USP34, MLLT6, GAPDH, PLAGL2, MAFG, WDFY2, BRD2, KLF13, ZNF121, AFF4, SON, SRSF4, VCP, RNF4, ARF4, HIVEP1, POFUT2, DUSP6 |

**Supplementary Table S4.** Biological processes and their related mRNAs in Normal-MCI subnetwork.

| Term | Count | PValue | Genes |
| --- | --- | --- | --- |
| GO:0010608~posttranscriptional regulation of gene expression | 14 | 3.01E-05 | ACO1, MKNK1, EIF4G1, EIF4G3, PPP1CA, MYD88, EIF3B, DGCR8, PSME1, MAPK14, KHSRP, NCOR1, EIF2B4, TOB1 |
| GO:0006417~regulation of translation | 11 | 1.65E-04 | EIF4G1, EIF4G3, PPP1CA, EIF3B, DGCR8, ACO1, KHSRP, MKNK1, NCOR1, EIF2B4, TOB1 |
| GO:0034248~regulation of cellular amide metabolic process | 11 | 3.29E-04 | EIF4G1, EIF4G3, PPP1CA, EIF3B, DGCR8, ACO1, KHSRP, MKNK1, NCOR1, EIF2B4, TOB1 |
| GO:0032268~regulation of cellular protein metabolic process | 31 | 7.70E-04 | MKNK1, AKAP13, NFKB1, DAB2, EIF3B, MYD88, DGCR8, MSN, EIF2B4, DVL3, DBNL, LAMTOR1, ACO1, MADD, MYH9, RHBDD1, EIF4G1, MAP4K4, EIF4G3, PPP1CA, EP300, PSME1, VCP, PHF1, MAPK14, KHSRP, CALM3, SEMA4D, PCSK1N, NCOR1, TOB1 |
| GO:0051246~regulation of protein metabolic process | 32 | 0.001118349 | MKNK1, AKAP13, NFKB1, DAB2, EIF3B, MYD88, DGCR8, MSN, EIF2B4, DVL3, DBNL, STX5, LAMTOR1, ACO1, MADD, MYH9, RHBDD1, EIF4G1, MAP4K4, EIF4G3, PPP1CA, EP300, PSME1, VCP, PHF1, MAPK14, KHSRP, CALM3, SEMA4D, PCSK1N, NCOR1, TOB1 |
| GO:0051403~stress-activated MAPK cascade | 8 | 0.001689304 | MAP4K4, DVL3, DBNL, SH2D3C, MYD88, MAPK14, NFKB1, NCOR1 |
| GO:0043547~positive regulation of GTPase activity | 13 | 0.001804823 | DVL3, ABR, LAMTOR1, MADD, ARHGEF18, AKAP13, SH2D3C, RCC2, SOS2, ARHGAP1, CALM3, SEMA4D, EIF2B4 |
| GO:0044267~cellular protein metabolic process | 51 | 0.001841879 | ARSD, COPS6, NFKB1, ST3GAL1, FNTB, DAB2, MYD88, DGCR8, MSN, EIF2B4, DBNL, MCRS1, ACO1, MADD, ZDHHC8, RHBDD1, MYH9, EIF4G1, SH2D3C, MAP4K4, PPP1CA, EIF4G3, EP300, DCAF7, PSME1, SLC25A34, HUWE1, KHSRP, DHPS, SEMA4D, UGGT1, FKBP5, MKNK1, AKAP13, PXK, RPA1, EIF3B, STK19, DVL3, LAMTOR1, KCTD2, ATXN7L3, MEF2D, SAP130, VCP, PHF1, MAPK14, CALM3, PCSK1N, NCOR1, TOB1 |
| GO:0031098~stress-activated protein kinase signaling cascade | 8 | 0.002220962 | MAP4K4, DVL3, DBNL, SH2D3C, MYD88, MAPK14, NFKB1, NCOR1 |
| GO:0009967~positive regulation of signal transduction | 21 | 0.002419926 | DVL3, DBNL, LAMTOR1, MADD, AKAP13, NFKB1, TLR5, RRAGC, SH2D3C, PPP1CA, DAB2, MYD88, PSME1, MAPK14, ARHGAP1, SOS2, CALM3, SEMA4D, TRIP6, LFNG, TOB1 |
| GO:0051641~cellular localization | 31 | 0.002757723 | AP1M1, HK1, AKAP13, VPS33A, RRAGC, TUBB, DAB2, DDX19A, GOLGA1, MSN, EHD1, VPS39, DVL3, STX5, MCRS1, LAMTOR1, ZDHHC8, RNPS1, MYH9, RHBDD1, MAP4K4, RCC2, VCP, HUWE1, MAPK14, CALM3, SYTL3, AP4B1, TRIP6, ALKBH5, TOB1 |
| GO:0006446~regulation of translational initiation | 5 | 0.002863706 | EIF4G1, EIF4G3, PPP1CA, EIF3B, EIF2B4 |
| GO:0033036~macromolecule localization | 33 | 0.003557863 | AP1M1, HK1, NFKB1, TLR5, VPS33A, RRAGC, DAB2, DDX19A, GOLGA1, APBA3, MSN, EHD1, VPS39, DVL3, STX5, MCRS1, LAMTOR1, ZDHHC8, RNPS1, MYH9, RHBDD1, MAP4K4, RCC2, VCP, HUWE1, MAPK14, KHSRP, SYTL3, AP4B1, TRIP6, NCOR1, ALKBH5, TOB1 |
| GO:0043087~regulation of GTPase activity | 13 | 0.00364831 | DVL3, ABR, LAMTOR1, MADD, ARHGEF18, AKAP13, SH2D3C, RCC2, SOS2, ARHGAP1, CALM3, SEMA4D, EIF2B4 |
| GO:0046907~intracellular transport | 22 | 0.003878069 | STX5, AP1M1, ZDHHC8, AKAP13, RNPS1, RHBDD1, VPS33A, TUBB, DAB2, VCP, HUWE1, DDX19A, MAPK14, GOLGA1, SYTL3, MSN, TRIP6, AP4B1, EHD1, ALKBH5, VPS39, TOB1 |
| GO:0006807~nitrogen compound metabolic process | 65 | 0.004104923 | ARSD, PTGES2, BTD, CNDP2, COPS6, NFKB1, TLR5, ST3GAL1, DAB2, MYD88, DGCR8, SND1, CHST14, SLC22A4, EIF2B4, TFIP11, SSBP3, MCRS1, PDXK, ACO1, GMEB2, EIF4G1, PPP1CA, EIF4G3, EP300, HUWE1, PSME1, SLC25A34, KHSRP, CELF2, DHPS, VGLL4, SEMA4D, UGGT1, APEX2, ALDOC, MKNK1, HK1, RRAGC, RPA1, EIF3B, DDX19A, PRPF8, GATAD2A, CSDE1, DVL3, TAF6, CREB5, RNPS1, ATXN7L3, QRICH1, SDHA, MEF2D, SAP130, PHF1, VCP, HNRNPUL1, MAPK14, MBOAT1, CALM3, TRIP6, PCSK1N, NCOR1, ALKBH5, TOB1 |
| GO:0007264~small GTPase mediated signal transduction | 11 | 0.004265117 | DBNL, SH2D3C, ABR, MADD, ARHGEF18, MAPK14, ARHGAP1, SOS2, AKAP13, RABL2B, RRAGC |
| GO:0043603~cellular amide metabolic process | 16 | 0.004444637 | CNDP2, ACO1, MKNK1, EIF4G1, ST3GAL1, EIF4G3, PPP1CA, EIF3B, DGCR8, SLC25A34, KHSRP, DHPS, PCSK1N, NCOR1, EIF2B4, TOB1 |
| GO:0006082~organic acid metabolic process | 15 | 0.004912257 | SDHA, ST3GAL1, PTGES2, BTD, CNDP2, ACO1, MAPK14, ALDOC, CS, FASN, ECHDC2, HK1, DHPS, NCOR1, EIF2B4 |
| GO:1903337~positive regulation of vacuolar transport | 3 | 0.004923063 | DAB2, MSN, EHD1 |
| GO:0051345~positive regulation of hydrolase activity | 15 | 0.00540267 | DVL3, ABR, LAMTOR1, MADD, ARHGEF18, AKAP13, SH2D3C, VCP, RCC2, PSME1, SOS2, ARHGAP1, CALM3, SEMA4D, EIF2B4 |
| GO:0006091~generation of precursor metabolites and energy | 9 | 0.005583161 | SDHA, PPP1CA, VCP, ACO1, ALDOC, CS, CALM3, HK1, NCOR1 |
| GO:0044237~cellular metabolic process | 87 | 0.005644809 | ARSD, PTGES2, BTD, CNDP2, COPS6, ECHDC2, NFKB1, TLR5, ST3GAL1, FNTB, DAB2, MYD88, DGCR8, SND1, CHST14, SLC22A4, MSN, EIF2B4, TFIP11, DBNL, SSBP3, MCRS1, PDXK, MADD, ACO1, GMEB2, ZDHHC8, RHBDD1, MYH9, EIF4G1, MAP4K4, SH2D3C, PPP1CA, EIF4G3, DCAF7, EP300, HUWE1, PSME1, SLC25A34, KHSRP, CELF2, DHPS, INPP4A, SEMA4D, VGLL4, UGGT1, ADD1, PGS1, APEX2, FKBP5, ALDOC, HK1, AKAP13, MKNK1, PXK, RRAGC, RPA1, EIF3B, DDX19A, PRPF8, GATAD2A, CSDE1, FASN, STK19, DVL3, TAF6, LAMTOR1, CS, CREB5, RNPS1, KCTD2, ATXN7L3, QRICH1, SDHA, MEF2D, SAP130, PHF1, VCP, HNRNPUL1, MAPK14, MBOAT1, CALM3, TRIP6, PCSK1N, NCOR1, ALKBH5, TOB1 |
| GO:0043604~amide biosynthetic process | 13 | 0.005993037 | CNDP2, ST3GAL1, EIF4G1, EIF4G3, PPP1CA, EIF3B, SLC25A34, DGCR8, KHSRP, DHPS, NCOR1, EIF2B4, TOB1 |
| GO:0034641~cellular nitrogen compound metabolic process | 61 | 0.00615247 | PTGES2, CNDP2, COPS6, NFKB1, TLR5, ST3GAL1, DAB2, MYD88, DGCR8, SND1, SLC22A4, EIF2B4, TFIP11, MCRS1, SSBP3, PDXK, ACO1, GMEB2, EIF4G1, PPP1CA, EIF4G3, EP300, HUWE1, PSME1, SLC25A34, KHSRP, CELF2, DHPS, VGLL4, SEMA4D, UGGT1, APEX2, ALDOC, MKNK1, HK1, RRAGC, RPA1, EIF3B, DDX19A, PRPF8, GATAD2A, CSDE1, DVL3, TAF6, CREB5, RNPS1, ATXN7L3, QRICH1, SDHA, MEF2D, SAP130, PHF1, VCP, HNRNPUL1, MAPK14, CALM3, PCSK1N, TRIP6, NCOR1, ALKBH5, TOB1 |
| GO:0033554~cellular response to stress | 23 | 0.006315938 | TFIP11, DVL3, DBNL, MCRS1, APEX2, COPS6, NFKB1, RHBDD1, RRAGC, RPA1, MAP4K4, SH2D3C, PPP1CA, EP300, MYD88, VCP, HUWE1, PHF1, MAPK14, NCOR1, UGGT1, EIF2B4, ADD1 |
| GO:0010647~positive regulation of cell communication | 21 | 0.006321207 | DVL3, DBNL, LAMTOR1, MADD, AKAP13, NFKB1, TLR5, RRAGC, SH2D3C, PPP1CA, DAB2, MYD88, PSME1, MAPK14, ARHGAP1, SOS2, CALM3, SEMA4D, TRIP6, LFNG, TOB1 |
| GO:0000165~MAPK cascade | 14 | 0.006607553 | DVL3, DBNL, LAMTOR1, MADD, AKAP13, NFKB1, SH2D3C, MAP4K4, MEF2D, MYD88, PSME1, MAPK14, CALM3, NCOR1 |
| GO:0051649~establishment of localization in the cell | 25 | 0.00664889 | STX5, AP1M1, ZDHHC8, AKAP13, RNPS1, MYH9, RHBDD1, VPS33A, MAP4K4, TUBB, DAB2, HUWE1, VCP, DDX19A, MAPK14, GOLGA1, CALM3, MSN, SYTL3, TRIP6, EHD1, AP4B1, ALKBH5, VPS39, TOB1 |
| GO:0023056~positive regulation of signaling | 21 | 0.006682939 | DVL3, DBNL, LAMTOR1, MADD, AKAP13, NFKB1, TLR5, RRAGC, SH2D3C, PPP1CA, DAB2, MYD88, PSME1, MAPK14, ARHGAP1, SOS2, CALM3, SEMA4D, TRIP6, LFNG, TOB1 |
| GO:0019538~protein metabolic process | 53 | 0.006726759 | ARSD, COPS6, CNDP2, NFKB1, ST3GAL1, FNTB, DAB2, MYD88, DGCR8, MSN, EIF2B4, STX5, DBNL, MCRS1, MADD, ACO1, ZDHHC8, RHBDD1, MYH9, EIF4G1, MAP4K4, SH2D3C, PPP1CA, EIF4G3, EP300, DCAF7, PSME1, SLC25A34, HUWE1, KHSRP, DHPS, SEMA4D, UGGT1, FKBP5, MKNK1, AKAP13, PXK, RPA1, EIF3B, STK19, DVL3, LAMTOR1, KCTD2, ATXN7L3, MEF2D, SAP130, VCP, PHF1, MAPK14, CALM3, PCSK1N, NCOR1, TOB1 |
| GO:0043043~peptide biosynthetic process | 12 | 0.007767913 | EIF4G1, EIF4G3, PPP1CA, EIF3B, DGCR8, SLC25A34, CNDP2, KHSRP, DHPS, NCOR1, EIF2B4, TOB1 |
| GO:0007265~Ras protein signal transduction | 8 | 0.007783709 | DBNL, ABR, MADD, ARHGEF18, MAPK14, ARHGAP1, SOS2, AKAP13 |
| GO:0030220~platelet formation | 3 | 0.007870765 | EP300, MYH9, VPS33A |
| GO:0008152~metabolic process | 92 | 0.008431236 | ARSD, PTGES2, BTD, CNDP2, COPS6, ECHDC2, ECHDC3, NFKB1, TLR5, ST3GAL1, FNTB, DAB2, MYD88, DGCR8, SND1, CHST14, SLC22A4, MSN, EIF2B4, TFIP11, DBNL, STX5, SSBP3, MCRS1, PDXK, MADD, ACO1, GMEB2, ZDHHC8, RHBDD1, MYH9, EIF4G1, MAP4K4, SH2D3C, EIF4G3, PPP1CA, DCAF7, EP300, HUWE1, SLC25A34, PSME1, KHSRP, CELF2, DHPS, INPP4A, SEMA4D, VGLL4, UGGT1, ADD1, PGS1, APEX2, FKBP5, ALDOC, HK1, AKAP13, MKNK1, PXK, RRAGC, RPA1, EIF3B, DDX19A, PRPF8, GATAD2A, APBA3, CSDE1, FASN, STK19, DVL3, TAF6, LAMTOR1, PYROXD2, CS, CREB5, RNPS1, KCTD2, ATXN7L3, QRICH1, SDHA, MEF2D, METTL13, SAP130, PHF1, VCP, HNRNPUL1, MAPK14, MBOAT1, CALM3, TRIP6, PCSK1N, NCOR1, ALKBH5, TOB1 |
| GO:0034613~cellular protein localization | 21 | 0.008585038 | STX5, MCRS1, AP1M1, LAMTOR1, ZDHHC8, HK1, RHBDD1, RRAGC, DAB2, RCC2, VCP, HUWE1, MAPK14, GOLGA1, SYTL3, MSN, TRIP6, AP4B1, EHD1, VPS39, TOB1 |
| GO:0036344~platelet morphogenesis | 3 | 0.008705043 | EP300, MYH9, VPS33A |
| GO:0043393~regulation of protein binding | 6 | 0.008750442 | TFIP11, DAB2, PPP1CA, EP300, LFNG, ADD1 |
| GO:0016197~endosomal transport | 7 | 0.00879957 | STX5, DAB2, AP1M1, MSN, EHD1, VPS33A, VPS39 |
| GO:0048522~positive regulation of cellular process | 47 | 0.008911656 | PTGES2, AKAP13, NFKB1, TLR5, RRAGC, RPA1, FNTB, DAB2, MYD88, SOS2, ARHGAP1, FASN, MSN, EHD1, LFNG, DVL3, DBNL, SSBP3, ABR, LAMTOR1, MADD, ZDHHC8, ARHGEF18, CREB5, RNPS1, RHBDD1, MYH9, ATXN7L3, EIF4G1, SH2D3C, MEF2D, PPP1CA, EP300, RCC2, PHF1, HUWE1, VCP, PSME1, MAPK14, KHSRP, CALM3, DHPS, SEMA4D, TRIP6, CYB5D2, NCOR1, TOB1 |
| GO:0023014~signal transduction by protein phosphorylation | 14 | 0.009237875 | DVL3, DBNL, LAMTOR1, MADD, AKAP13, NFKB1, SH2D3C, MAP4K4, MEF2D, MYD88, PSME1, MAPK14, CALM3, NCOR1 |
| GO:0070727~cellular macromolecule localization | 21 | 0.009350133 | STX5, MCRS1, AP1M1, LAMTOR1, ZDHHC8, HK1, RHBDD1, RRAGC, DAB2, RCC2, VCP, HUWE1, MAPK14, GOLGA1, SYTL3, MSN, TRIP6, AP4B1, EHD1, VPS39, TOB1 |
| GO:0009896~positive regulation of catabolic process | 8 | 0.009571128 | STX5, DAB2, VCP, PSME1, KHSRP, MSN, RHBDD1, TOB1 |
| GO:0071310~cellular response to organic substance | 26 | 0.00988557 | NFKB1, TLR5, RRAGC, DAB2, MYD88, DGCR8, IL10RB, PRPF8, FASN, MSN, EHD1, LAMTOR1, MADD, ARHGEF18, HLA-B, RHBDD1, MAP4K4, EP300, PSME1, VCP, MAPK14, KHSRP, NCOR1, UGGT1, TOB1, ADD1 |

**Supplementary Table S5.** Biological processes and their related mRNAs in MCI-AD subnetwork.

| Term | Count | PValue | Genes |
| --- | --- | --- | --- |
| GO:1903311~regulation of mRNA metabolic process | 10 | 1.04E-06 | ZFP36, SON, SRSF4, HNRNPK, BTG2, RBM5, ZC3H12A, SNRNP70, NELFE, TNRC6B |
| GO:0016071~mRNA metabolic process | 17 | 7.60E-05 | ZFP36, TFIP11, RALY, RBM5, SF3B2, SON, SRSF4, AKAP17A, HNRNPK, BTG2, HNRNPUL1, PCBP1, ZC3H12A, SNRNP70, TNRC6B, NELFE, DXO |
| GO:0016032~viral process | 20 | 1.96E-04 | ZFP36, AP2S1, VIM, NFKBIA, HLA-B, USF2, SF3B2, AP2B1, HNRNPK, VCP, CD93, HNRNPUL1, CXCR4, IL10RB, PCBP1, UBC, ZC3H12A, E4F1, NELFE, TBC1D20 |
| GO:0044764~multi-organism cellular process | 20 | 2.15E-04 | ZFP36, AP2S1, VIM, NFKBIA, HLA-B, USF2, SF3B2, AP2B1, HNRNPK, VCP, CD93, HNRNPUL1, CXCR4, IL10RB, PCBP1, UBC, ZC3H12A, E4F1, NELFE, TBC1D20 |
| GO:0016192~vesicle-mediated transport | 26 | 2.28E-04 | ALDOA, CYTH1, AP2S1, MAPKAPK3, TFG, CANX, TIMP1, CTNNB1, AP2B1, HNRNPK, RAC2, CD93, STX16, GOLGA1, DNAJC5, TMEM79, STX5, PHACTR2, XKR8, RAB11FIP3, VCP, ATP2A2, ARF4, CTSC, VAMP1, TBC1D20 |
| GO:0044419~interspecies interaction between organisms | 20 | 2.96E-04 | ZFP36, AP2S1, VIM, NFKBIA, HLA-B, USF2, SF3B2, AP2B1, HNRNPK, VCP, CD93, HNRNPUL1, CXCR4, IL10RB, PCBP1, UBC, ZC3H12A, E4F1, NELFE, TBC1D20 |
| GO:0044403~symbiosis, encompassing mutualism through parasitism | 20 | 2.96E-04 | ZFP36, AP2S1, VIM, NFKBIA, HLA-B, USF2, SF3B2, AP2B1, HNRNPK, VCP, CD93, HNRNPUL1, CXCR4, IL10RB, PCBP1, UBC, ZC3H12A, E4F1, NELFE, TBC1D20 |
| GO:1903313~positive regulation of mRNA metabolic process | 5 | 6.85E-04 | ZFP36, BTG2, ZC3H12A, SNRNP70, TNRC6B |
| GO:0061014~positive regulation of mRNA catabolic process | 4 | 0.001081488 | ZFP36, BTG2, ZC3H12A, TNRC6B |
| GO:0061615~glycolytic process through fructose-6-phosphate | 4 | 0.001209719 | ALDOA, GALK1, ALDOC, GAPDH |
| GO:0061620~glycolytic process through glucose-6-phosphate | 4 | 0.001209719 | ALDOA, GALK1, ALDOC, GAPDH |
| GO:0006397~mRNA processing | 12 | 0.001235662 | RALY, TFIP11, SON, SRSF4, HNRNPK, AKAP17A, HNRNPUL1, PCBP1, RBM5, SNRNP70, NELFE, SF3B2 |
| GO:0008380~RNA splicing | 11 | 0.001244105 | RALY, TFIP11, SON, SRSF4, HNRNPK, AKAP17A, HNRNPUL1, PCBP1, RBM5, SNRNP70, SF3B2 |
| GO:0009896~positive regulation of catabolic process | 10 | 0.001277015 | ZFP36, CREBRF, STX5, BTG2, VCP, UBC, ZC3H12A, CTSC, TNRC6B, TMEM259 |
| GO:0050684~regulation of mRNA processing | 6 | 0.001373689 | SON, SRSF4, HNRNPK, RBM5, SNRNP70, NELFE |
| GO:0061013~regulation of mRNA catabolic process | 4 | 0.001493997 | ZFP36, BTG2, ZC3H12A, TNRC6B |
| GO:0043484~regulation of RNA splicing | 6 | 0.001691583 | SON, SRSF4, HNRNPK, AKAP17A, RBM5, SNRNP70 |
| GO:0006096~glycolytic process | 5 | 0.001704893 | ALDOA, GALK1, ADPGK, ALDOC, GAPDH |
| GO:0006757~ATP generation from ADP | 5 | 0.001803963 | ALDOA, GALK1, ADPGK, ALDOC, GAPDH |
| GO:0051641~cellular localization | 34 | 0.002027445 | CREBRF, AP2S1, PPFIA1, NFKBIA, TFG, CTCF, CANX, CTNNB1, HSPA1L, AP2B1, RAC2, DDX19A, STX16, GOLGA1, ZC3H12A, DNAJC5, SPIDR, TUBA1C, PLAGL2, ZFP36, STX5, TWF2, LAMTOR1, BANP, SRSF4, RAB11FIP3, ATP2A2, VCP, ARF4, UBL4A, UBC, CTSC, RAB12, TBC1D20 |
| GO:0010604~positive regulation of macromolecule metabolic process | 37 | 0.002034043 | KMT2E, CREBRF, TAF1C, VIM, MAPKAPK3, NFKBIA, CTCF, TMEM259, CTNNB1, NOD2, HNRNPK, CXCR4, PCBP1, MTCH1, ZC3H12A, SPIDR, SNRNP70, TNRC6B, PLAGL2, ZFP36, MAFG, STX5, WDFY2, LAMTOR1, KLF13, BANP, IL6R, USF2, BTG2, RNF4, VCP, ARF4, UBC, CTSC, NELFE, TBX19, DUSP6 |
| GO:0060255~regulation of macromolecule metabolic process | 63 | 0.002204537 | RALY, CREBRF, TAF1C, RBM5, CTCF, CTNNB1, NOD2, AKAP17A, CXCR4, PCBP1, RNF38, E4F1, DXO, ELMSAN1, TFIP11, ZFP36, STX5, ANKRA2, BANP, IL6R, PRKCB, BTG2, UBC, CTSC, LCOR, TBX19, KMT2E, HIST1H2AC, VIM, MAPKAPK3, NFKBIA, TMEM259, TIMP1, HNRNPK, RAC2, MTCH1, APBA3, ZC3H12A, SPIDR, SNRNP70, USP34, TNRC6B, MLLT6, GAPDH, PLAGL2, MAFG, WDFY2, BRD2, LAMTOR1, KLF13, ZNF121, AFF4, USF2, SON, SRSF4, VCP, RNF4, HNRNPUL1, ARF4, POFUT2, HIVEP1, NELFE, DUSP6 |
| GO:0048024~regulation of mRNA splicing, via spliceosome | 5 | 0.002240269 | SON, SRSF4, HNRNPK, RBM5, SNRNP70 |
| GO:0009894~regulation of catabolic process | 12 | 0.00227662 | ZFP36, CREBRF, STX5, BTG2, VCP, UBC, ZC3H12A, CTSC, BANP, TNRC6B, TMEM259, TIMP1 |
| GO:0000377~RNA splicing, via transesterification reactions with bulged adenosine as the nucleophile | 9 | 0.00252124 | RALY, TFIP11, SRSF4, HNRNPK, HNRNPUL1, PCBP1, RBM5, SNRNP70, SF3B2 |
| GO:0000398~mRNA splicing, via spliceosome | 9 | 0.00252124 | RALY, TFIP11, SRSF4, HNRNPK, HNRNPUL1, PCBP1, RBM5, SNRNP70, SF3B2 |
| GO:0009057~macromolecule catabolic process | 20 | 0.00266819 | ZFP36, CREBRF, STX5, BANP, KCTD2, CTSS, TMEM259, TIMP1, CTNNB1, VCP, RNF4, BTG2, RNF38, UBC, ZC3H12A, CTSC, RAB12, TNRC6B, USP34, DXO |
| GO:0000375~RNA splicing, via transesterification reactions | 9 | 0.002735539 | RALY, TFIP11, SRSF4, HNRNPK, HNRNPUL1, PCBP1, RBM5, SNRNP70, SF3B2 |
| GO:0046031~ADP metabolic process | 5 | 0.00274438 | ALDOA, GALK1, ADPGK, ALDOC, GAPDH |
| GO:0046496~nicotinamide nucleotide metabolic process | 6 | 0.002867553 | ALDOA, GALK1, VCP, ADPGK, ALDOC, GAPDH |
| GO:0019362~pyridine nucleotide metabolic process | 6 | 0.002867553 | ALDOA, GALK1, VCP, ADPGK, ALDOC, GAPDH |
| GO:0006734~NADH metabolic process | 4 | 0.003034792 | ALDOA, VCP, ALDOC, GAPDH |
| GO:0051650~establishment of vesicle localization | 8 | 0.003037836 | STX5, ATP2A2, TFG, CTSC, DNAJC5, TBC1D20, CANX, CTNNB1 |
| GO:0060213~positive regulation of nuclear-transcribed mRNA poly(A) tail shortening | 3 | 0.003219958 | ZFP36, BTG2, TNRC6B |
| GO:0060211~regulation of nuclear-transcribed mRNA poly(A) tail shortening | 3 | 0.003219958 | ZFP36, BTG2, TNRC6B |
| GO:0051254~positive regulation of RNA metabolic process | 22 | 0.003389209 | KMT2E, ZFP36, MAFG, KLF13, NFKBIA, BANP, CTCF, USF2, CTNNB1, NOD2, HNRNPK, RNF4, BTG2, PCBP1, ARF4, UBC, ZC3H12A, SNRNP70, TNRC6B, NELFE, TBX19, PLAGL2 |
| GO:0072524~pyridine-containing compound metabolic process | 6 | 0.00376279 | ALDOA, GALK1, VCP, ADPGK, ALDOC, GAPDH |
| GO:0051648~vesicle localization | 8 | 0.003962885 | STX5, ATP2A2, TFG, CTSC, DNAJC5, TBC1D20, CANX, CTNNB1 |
| GO:0006810~transport | 52 | 0.004286929 | CREBRF, AP2S1, CANX, SLC35A2, CTNNB1, AP2B1, NOD2, CD93, GOLGA1, DNAJC5, TUBA1C, ZFP36, STX5, CTSS, HERC1, PRKCB, RAB11FIP3, UBL4A, UBC, CTSC, RAB12, SLC38A1, VAMP1, ALDOA, CYTH1, PPFIA1, MAPKAPK3, TFG, NFKBIA, TIMP1, HSPA1L, HNRNPK, RAC2, DDX19A, MTCH1, STX16, APBA3, ZC3H12A, TMEM79, PLAGL2, SLC39A1, PHACTR2, LAMTOR1, XKR8, USF2, SRSF4, ATP2A2, VCP, ARF4, POFUT2, CLCN6, TBC1D20 |
| GO:0006165~nucleoside diphosphate phosphorylation | 5 | 0.00433334 | ALDOA, GALK1, ADPGK, ALDOC, GAPDH |
| GO:0051252~regulation of RNA metabolic process | 44 | 0.004506243 | KMT2E, RALY, CREBRF, HIST1H2AC, TAF1C, RBM5, NFKBIA, CTCF, CTNNB1, NOD2, HNRNPK, AKAP17A, PCBP1, RNF38, ZC3H12A, E4F1, SNRNP70, USP34, TNRC6B, MLLT6, PLAGL2, ELMSAN1, TFIP11, ZFP36, MAFG, BRD2, ANKRA2, KLF13, ZNF121, AFF4, BANP, USF2, PRKCB, SON, SRSF4, BTG2, RNF4, HNRNPUL1, ARF4, UBC, HIVEP1, LCOR, NELFE, TBX19 |
| GO:0051234~establishment of localization | 53 | 0.004510033 | CREBRF, AP2S1, CANX, SLC35A2, CTNNB1, AP2B1, NOD2, CD93, GOLGA1, DNAJC5, TUBA1C, ZFP36, STX5, CTSS, HERC1, PRKCB, RAB11FIP3, UBL4A, UBC, CTSC, RAB12, SLC38A1, VAMP1, ALDOA, CYTH1, PPFIA1, MAPKAPK3, TFG, NFKBIA, TIMP1, HSPA1L, HNRNPK, RAC2, DDX19A, MTCH1, STX16, APBA3, ZC3H12A, SPIDR, TMEM79, PLAGL2, SLC39A1, PHACTR2, LAMTOR1, XKR8, USF2, SRSF4, ATP2A2, VCP, ARF4, POFUT2, CLCN6, TBC1D20 |
| GO:0006915~apoptotic process | 25 | 0.004620485 | ZFP36, XKR8, RBM5, NFKBIA, IL6R, ANXA5, DIDO1, PRKCB, TIMP1, CTNNB1, SON, NOD2, HNRNPK, VCP, BTG2, CXCR4, MTCH1, ARF4, UBC, ZC3H12A, CTSC, DNAJC5, GAPDH, DUSP6, PLAGL2 |
| GO:0030163~protein catabolic process | 15 | 0.004678055 | CREBRF, STX5, BANP, KCTD2, CTSS, TMEM259, TIMP1, CTNNB1, VCP, RNF4, RNF38, UBC, RAB12, CTSC, USP34 |
| GO:0012501~programmed cell death | 26 | 0.004790691 | RBM5, NFKBIA, DIDO1, CTNNB1, TIMP1, NOD2, HNRNPK, CXCR4, MTCH1, ZC3H12A, DNAJC5, GAPDH, TMEM79, PLAGL2, ZFP36, XKR8, IL6R, ANXA5, PRKCB, SON, BTG2, VCP, ARF4, UBC, CTSC, DUSP6 |
| GO:0006733~oxidoreduction coenzyme metabolic process | 6 | 0.0048628 | ALDOA, GALK1, VCP, ADPGK, ALDOC, GAPDH |
| GO:0010468~regulation of gene expression | 49 | 0.004880556 | RALY, CREBRF, TAF1C, RBM5, CTCF, CTNNB1, NOD2, AKAP17A, PCBP1, RNF38, E4F1, DXO, ELMSAN1, TFIP11, ZFP36, ANKRA2, BANP, PRKCB, BTG2, UBC, LCOR, TBX19, KMT2E, HIST1H2AC, VIM, NFKBIA, HNRNPK, APBA3, ZC3H12A, SNRNP70, USP34, TNRC6B, MLLT6, GAPDH, PLAGL2, MAFG, BRD2, KLF13, ZNF121, AFF4, USF2, SON, SRSF4, RNF4, HNRNPUL1, ARF4, HIVEP1, POFUT2, NELFE |
| GO:0008219~cell death | 27 | 0.004960869 | RBM5, NFKBIA, TMEM259, DIDO1, CTNNB1, TIMP1, NOD2, HNRNPK, CXCR4, MTCH1, ZC3H12A, DNAJC5, GAPDH, TMEM79, PLAGL2, ZFP36, XKR8, IL6R, ANXA5, PRKCB, SON, BTG2, VCP, ARF4, UBC, CTSC, DUSP6 |
| GO:0045935~positive regulation of nucleobase-containing compound metabolic process | 24 | 0.004968732 | KMT2E, ZFP36, MAFG, KLF13, NFKBIA, BANP, CTCF, USF2, CTNNB1, NOD2, HNRNPK, VCP, RNF4, BTG2, PCBP1, ARF4, UBC, ZC3H12A, SPIDR, SNRNP70, TNRC6B, NELFE, TBX19, PLAGL2 |
| GO:0009135~purine nucleoside diphosphate metabolic process | 5 | 0.005321261 | ALDOA, GALK1, ADPGK, ALDOC, GAPDH |
| GO:0009179~purine ribonucleoside diphosphate metabolic process | 5 | 0.005321261 | ALDOA, GALK1, ADPGK, ALDOC, GAPDH |
| GO:0046939~nucleotide phosphorylation | 5 | 0.005535439 | ALDOA, GALK1, ADPGK, ALDOC, GAPDH |
| GO:0009185~ribonucleoside diphosphate metabolic process | 5 | 0.005755304 | ALDOA, GALK1, ADPGK, ALDOC, GAPDH |
| GO:0006139~nucleobase-containing compound metabolic process | 61 | 0.005804227 | RALY, CREBRF, TAF1C, RBM5, CTCF, CTNNB1, NOD2, AKAP17A, PCBP1, RNF38, E4F1, DXO, ELMSAN1, MRI1, TFIP11, ZFP36, ANKRA2, POLG, BANP, PRKCB, BTG2, UBC, LCOR, TBX19, KMT2E, ALDOA, HIST1H2AC, ADPGK, ALDOC, UPP1, NFKBIA, HDGFRP2, DIDO1, SF3B2, ZFC3H1, RPA1, GALK1, HNRNPK, DDX19A, ZC3H12A, SPIDR, SNRNP70, USP34, TNRC6B, MLLT6, GAPDH, PLAGL2, MAFG, BRD2, KLF13, ZNF121, AFF4, USF2, SON, SRSF4, VCP, RNF4, HNRNPUL1, ARF4, HIVEP1, NELFE |
| GO:1901575~organic substance catabolic process | 26 | 0.006162885 | CREBRF, ALDOA, ADPGK, ALDOC, UPP1, TMEM259, CTNNB1, TIMP1, GALK1, RNF38, ZC3H12A, TNRC6B, USP34, DXO, GAPDH, ZFP36, STX5, BANP, CTSS, KCTD2, BTG2, RNF4, VCP, UBC, RAB12, CTSC |
| GO:0009893~positive regulation of metabolic process | 37 | 0.006266801 | KMT2E, CREBRF, TAF1C, VIM, MAPKAPK3, NFKBIA, CTCF, TMEM259, CTNNB1, NOD2, HNRNPK, CXCR4, PCBP1, MTCH1, ZC3H12A, SPIDR, SNRNP70, TNRC6B, PLAGL2, ZFP36, MAFG, STX5, WDFY2, LAMTOR1, KLF13, BANP, IL6R, USF2, BTG2, RNF4, VCP, ARF4, UBC, CTSC, NELFE, TBX19, DUSP6 |
| GO:0046907~intracellular transport | 23 | 0.006396817 | CREBRF, STX5, AP2S1, NFKBIA, TFG, CTNNB1, HSPA1L, AP2B1, SRSF4, RAC2, ATP2A2, VCP, DDX19A, STX16, ARF4, UBC, GOLGA1, ZC3H12A, CTSC, RAB12, TBC1D20, TUBA1C, PLAGL2 |
| GO:1900151~regulation of nuclear-transcribed mRNA catabolic process, deadenylation-dependent decay | 3 | 0.006847095 | ZFP36, BTG2, TNRC6B |
| GO:1900153~positive regulation of nuclear-transcribed mRNA catabolic process, deadenylation-dependent decay | 3 | 0.006847095 | ZFP36, BTG2, TNRC6B |
| GO:0006090~pyruvate metabolic process | 5 | 0.007197542 | ALDOA, GALK1, ADPGK, ALDOC, GAPDH |
| GO:0019219~regulation of nucleobase-containing compound metabolic process | 46 | 0.007852699 | KMT2E, RALY, CREBRF, HIST1H2AC, TAF1C, RBM5, NFKBIA, CTCF, CTNNB1, NOD2, HNRNPK, AKAP17A, PCBP1, RNF38, ZC3H12A, SPIDR, E4F1, SNRNP70, USP34, TNRC6B, MLLT6, PLAGL2, ELMSAN1, TFIP11, ZFP36, MAFG, BRD2, ANKRA2, KLF13, ZNF121, AFF4, BANP, USF2, PRKCB, SON, SRSF4, BTG2, RNF4, VCP, HNRNPUL1, ARF4, UBC, HIVEP1, LCOR, NELFE, TBX19 |
| GO:0048518~positive regulation of biological process | 56 | 0.008388381 | CREBRF, TAF1C, RBM5, CTCF, CTNNB1, NOD2, CXCR4, PCBP1, ZFP36, STX5, TWF2, CNPY3, BANP, HLA-B, IL6R, CTSS, PRKCB, BTG2, UBC, CTSC, RAB12, TBX19, KMT2E, VIM, MAPKAPK3, NFKBIA, TFG, TMEM259, HDGFRP2, TIMP1, RPA1, HSPA1L, HNRNPK, RAC2, MTCH1, ZC3H12A, SPIDR, SNRNP70, USP34, TNRC6B, TMEM79, PLAGL2, MAFG, WDFY2, LAMTOR1, KLF13, USF2, ATP2A2, VCP, RNF4, ARF4, JAK1, NELFE, CMTM3, TBC1D20, DUSP6 |
| GO:0006357~regulation of transcription from RNA polymerase II promoter | 25 | 0.008431812 | CREBRF, NFKBIA, CTCF, CTNNB1, NOD2, HNRNPK, PCBP1, ZC3H12A, E4F1, ELMSAN1, PLAGL2, MAFG, ZFP36, BRD2, ANKRA2, KLF13, USF2, PRKCB, BTG2, RNF4, ARF4, UBC, HIVEP1, NELFE, TBX19 |
| GO:0002221~pattern recognition receptor signaling pathway | 6 | 0.008438729 | NOD2, MAPKAPK3, CNPY3, UBC, NFKBIA, CTSS |
| GO:1901360~organic cyclic compound metabolic process | 63 | 0.009178598 | CREBRF, RALY, TAF1C, RBM5, CTCF, CTNNB1, NOD2, AKAP17A, PCBP1, RNF38, E4F1, DXO, LBR, ELMSAN1, MRI1, TFIP11, ZFP36, ANKRA2, POLG, BANP, PRKCB, BTG2, UBC, LCOR, TBX19, KMT2E, ALDOA, HIST1H2AC, ADPGK, ALDOC, UPP1, NFKBIA, HDGFRP2, DIDO1, SF3B2, ZFC3H1, RPA1, GALK1, HNRNPK, DDX19A, ZC3H12A, SPIDR, SNRNP70, USP34, TNRC6B, MLLT6, GAPDH, PLAGL2, MAFG, BRD2, LAMTOR1, KLF13, ZNF121, AFF4, USF2, SON, SRSF4, VCP, RNF4, HNRNPUL1, ARF4, HIVEP1, NELFE |
| GO:0019320~hexose catabolic process | 4 | 0.009248818 | ALDOA, GALK1, ALDOC, GAPDH |
| GO:0070987~error-free translesion synthesis | 3 | 0.009608163 | RPA1, VCP, UBC |
| GO:0046483~heterocycle metabolic process | 61 | 0.009643964 | RALY, CREBRF, TAF1C, RBM5, CTCF, CTNNB1, NOD2, AKAP17A, PCBP1, RNF38, E4F1, DXO, ELMSAN1, MRI1, TFIP11, ZFP36, ANKRA2, POLG, BANP, PRKCB, BTG2, UBC, LCOR, TBX19, KMT2E, ALDOA, HIST1H2AC, ADPGK, ALDOC, UPP1, NFKBIA, HDGFRP2, DIDO1, SF3B2, ZFC3H1, RPA1, GALK1, HNRNPK, DDX19A, ZC3H12A, SPIDR, SNRNP70, USP34, TNRC6B, MLLT6, GAPDH, PLAGL2, MAFG, BRD2, KLF13, ZNF121, AFF4, USF2, SON, SRSF4, VCP, RNF4, HNRNPUL1, ARF4, HIVEP1, NELFE |
| GO:0019318~hexose metabolic process | 7 | 0.009892833 | ALDOA, GALK1, ADPGK, ALDOC, POFUT2, SLC35A2, GAPDH |
| GO:0019222~regulation of metabolic process | 63 | 0.009997774 | RALY, CREBRF, TAF1C, RBM5, CTCF, CTNNB1, NOD2, AKAP17A, CXCR4, PCBP1, RNF38, E4F1, DXO, ELMSAN1, TFIP11, ZFP36, STX5, ANKRA2, BANP, IL6R, PRKCB, BTG2, UBC, CTSC, LCOR, TBX19, KMT2E, HIST1H2AC, VIM, MAPKAPK3, NFKBIA, TMEM259, TIMP1, HNRNPK, RAC2, MTCH1, APBA3, ZC3H12A, SPIDR, SNRNP70, USP34, TNRC6B, MLLT6, GAPDH, PLAGL2, MAFG, WDFY2, BRD2, LAMTOR1, KLF13, ZNF121, AFF4, USF2, SON, SRSF4, VCP, RNF4, HNRNPUL1, ARF4, POFUT2, HIVEP1, NELFE, DUSP6 |

**Supplementary Table S6.** Biological processes and their related mRNAs in Normal-AD subnetwork.

| Category | Term | P-value | miRNAs |
| --- | --- | --- | --- |
| Family | mir-15 family | 0.009691 | has-mir-15b, has-mir-16, has-mir-15a, has-mir-195 |
| Family | mir-17 family | 0.003159 | has-mir-20a, has-mir-93, has-mir-17, has-mir-18b, has-mir-106a, has-mir-20b, has-mir-18a, has-mir-106b |

**Supplementary Table S7.** Family of miRNAs in Normal-MCI subnetwork.

| Category | Term | Count | Percent | Fold | P-value | Bonferroni | FDR |
| --- | --- | --- | --- | --- | --- | --- | --- |
| Function | Apoptosis | 6 | 0.136364 | 3.222488 | 0.006442 | 0.6185 | 0.1522 |
| Function | Cell cycle related | 8 | 0.121212 | 2.864434 | 0.002869 | 0.2755 | 0.092 |
| Function | HIV latency | 4 | 0.190476 | 4.501253 | 0.008702 | 0.8354 | 0.1699 |
| Function | Hormones regulation | 8 | 0.129032 | 3.049236 | 0.001865 | 0.179 | 0.0644 |
| Function | Human embryonic stem cell (hESC) regulation | 10 | 0.117647 | 2.780186 | 0.000761 | 0.073 | 0.1139 |
| Function | cell proliferation(Hwang etal BJC2006) | 3 | 0.5 | 11.81579 | 0.001192 | 0.1144 | 0.0764 |
| Function | immune system(Xiao's Cell2009) | 4 | 0.222222 | 5.251462 | 0.004833 | 0.464 | 0.1206 |
| Function | onco-miRNAs | 5 | 0.16129 | 3.811545 | 0.006584 | 0.632 | 0.1478 |

**Supplementary Table S8.** The functionality of miRNAs in Normal-MCI subnetwork.

| Category | Term | P-value | miRNAs |
| --- | --- | --- | --- |
| Family | mir-17 family | 1.54E-06 | has-mir-20a, has-mir-106a, has-mir-93, has-mir-106b, has-mir-17, has-mir-18b, has-mir-20b, has-mir-18a |

**Supplementary Table S9.** Family of miRNAs in MCI-AD subnetwork.

| Category | Term | Count | Percent | Fold | P-value | Bonferroni | FDR |
| --- | --- | --- | --- | --- | --- | --- | --- |
| Function | Angiogenesis | 4 | 0.166667 | 4.677083 | 0.007454 | 0.6112 | 0.1154 |
| Function | HIV latency | 5 | 0.238095 | 6.681548 | 0.000427 | 0.035 | 0.0213 |
| Function | Hormones regulation | 9 | 0.145161 | 4.073589 | 5.5E-05 | 4.51E-03 | 6.18E-03 |
| Function | Human embryonic stem cell (hESC) regulation | 9 | 0.105882 | 2.971324 | 0.000765 | 0.0628 | 0.0191 |
| Function | cell proliferation(Hwang etal BJC2006) | 3 | 0.5 | 14.03125 | 0.000699 | 0.0574 | 0.0242 |
| Function | immune system(Xiao's Cell2009) | 6 | 0.333333 | 9.354167 | 1.07E-05 | 8.74E-04 | 2.39E-03 |
| Function | onco-miRNAs | 7 | 0.225806 | 6.336694 | 2.78E-05 | 2.28E-03 | 4.16E-03 |

**Supplementary Table S10.** The functionality of miRNAs in MCI-AD subnetwork.

| Category | Term | P-value | miRNAs |
| --- | --- | --- | --- |
| Family | mir-15 family | 0.009691 | has-mir-15b, has-mir-16, has-mir-15a, has-mir-195 |
| Family | mir-17 family | 0.000146 | has-mir-20a, has-mir-93, has-mir-106b, has-mir-17, has-mir-18b, has-mir-106a, has-mir-20b, has-mir-18a |

**Supplementary Table S11.** Family of miRNAs in Normal-AD subnetwork.

| Category | Term | Count | Percent | Fold | P-value | Bonferroni | FDR |
| --- | --- | --- | --- | --- | --- | --- | --- |
| Function | Apoptosis | 6 | 0.136364 | 3.222488 | 0.006442 | 0.5927 | 0.1071 |
| Function | HIV latency | 5 | 0.238095 | 5.626566 | 0.001035 | 0.0953 | 0.0465 |
| Function | Hormones regulation | 8 | 0.129032 | 3.049236 | 0.001865 | 0.1716 | 0.0381 |
| Function | Human embryonic stem cell (hESC) regulation | 11 | 0.129412 | 3.058204 | 0.000126 | 0.0116 | 0.0189 |
| Function | cell proliferation(Hwang etal BJC2006) | 3 | 0.5 | 11.81579 | 0.001192 | 0.1096 | 0.0412 |
| Function | immune system(Xiao's Cell2009) | 5 | 0.277778 | 6.564327 | 0.000473 | 0.0435 | 0.0354 |
| Function | onco-miRNAs | 6 | 0.193548 | 4.573854 | 0.00095 | 0.0874 | 0.0533 |

**Supplementary Table S12.** The functionality of miRNAs in Normal-AD subnetwork.

| The module names | Genes | Number of the members |
| --- | --- | --- |
| Elements included exclusively in "MCI-AD" | ABR, AKAP13, AP1M1, APEX2, ARHGAP1, ARSD, ATXN7L3, BTD, C16ORF70, C19ORF52, CELF2, COPS6, CREB5, CSDE1, CTAGE5, CYB5D2, DBNL, DVL3, ECHDC3, EHD1, EIF4G3, EP300, FKBP5, FLYWCH1, FNTB, GATAD2A, GMEB2, INPP4A, LY6G5B, MAP4K4, MAPK14, MAU2, MCTP2, MKNK1, MSN, MYD88, MYH9, NCOR1, PDXK, PGS1, PPP1CA, PXK, SAP130, SEMA4D, SH2D3C, SH3KBP1, SLC22A4, SLC25A34, SOS2, SZRD1, TLR5, TOB1, TUBB, TUBG1, UGGT1, VPS33A, ACO1, ADD1, ALKBH5, ANXA6, AP4B1, ARHGEF18, CALM3, CNDP2, CS, DAB2, DCAF7, DGCR8, DHPS, ECHDC2, EIF2B4, EIF3B, EIF4G1, FAM222B, FASN, HK1, HNRNPUL2, HUWE1, KHSRP, LAIR1, LFNG, MADD, MCRS1, MEF2D, METTL13, NFKB1, PCSK1N, PHF1, PRPF8, PSME1, PTGES2, PYROXD2, QRICH1, RABAC1, RCC2, RHBDD1, RNF26, RNPS1, RRAGC, SDHA, SMAGP, SND1, SPATA20, SPECC1L, SSBP3, ST3GAL1, STK19, SYTL3, TAF6, TRIP6, UNC45A, VGLL4, VPS39, ZDHHC8 | 114 |
| Elements in "CTL-MCI" and "CTL-AD" | ADPGK, AFF4, AGPAT3, AKAP17A, ALDOA, ANKHD1, ANKRA2, ANKRD11, ANXA5, ATP2A2, BRD2, CANX, CD93, CLCN6, CMTM3, CREBRF, CTCF, CTSC, CTSS, CYTH1, DCAF5, DIDO1, DUSP6, E4F1, ELMSAN1, HERC1, HIVEP1, HSPA1L, IK, KLF13, KMT2E, LCOR, LPP, MLLT6, MRI1, MTCH1, NOD2, PCBP1, PHACTR2, PHACTR4, POFUT2, POLG, PPFIA1, PRKCB, PRRC2C, RAB11FIP3, RAB12, RAC2, RALGAPA2, RASA2, RBM5, RNF4, SLC38A1, SNRNP70, SPIDR, SRSF4, STX16, TAF1C, TFG, TJAP1, TMEM259, TUBA1C, UBC, USP34, VAMP1, VIM, WDR91, ZC3H12A, ZFC3H1, ZFP36, ZNF121, ZNF598, ZNF839, ADAP1, ARF4, C2ORF68, CNPY3, CTNNB1, CXCR4, DNAJC5, GALK1, GAPDH, HIST1H2AC, HNRNPK, IL6R, JAK1, LBR, MAFG, MAPKAPK3, NFKBIA, PAQR4, PLAGL2, PLEKHB2, PPM1M, PRCC, RBM12, RDH5, RNF38, SF3B2, SLC39A1, SON, TAGAP, TBX19, TIMP1, UPP1, WDFY2, XKR8 | 107 |
| Elements in "CTL-MCI", "MCI-AD" and "CTL-AD" | GOLGA1, HLA-B, MBOAT1, RABL2B, ARMC7, IL10RB, STX5, TFIP11, VCP | 9 |
| Elements in "MCI-AD" and "CTL-AD" | ALDOC, APBA3, CHST14, DDX19A, HNRNPUL1, KCTD2, LAMTOR1, RPA1 | 8 |
| Elements included exclusively in "CTL-AD" | AP2B1, AP2S1, BANP, BTG2, C8ORF82, DXO, GLTSCR1, HDGFRP2, KLHL35, NELFE, NOMO1, OGFR, RALY, SLC35A2, TBC1D20, TMEM79, TNRC6B, TWF2, UBL4A, USF2, ZCCHC24 | 21 |

**Supplementary Table S13.** The names, related modules and the number of genes proposed as a biomarker by this study.

| The module names | miRNAs | Number of the members |
| --- | --- | --- |
| Element included exclusively in "CTL-MCI" | hsa-miR-98-5p | 1 |
| Elements included exclusively in "MCI-AD" | hsa-miR-4722-5p, hsa-miR-106a-5p, hsa-miR-4768-3p, hsa-miR-1827, hsa-miR-940, hsa-miR-30b-3p | 6 |
| Elements in "CTL-MCI" and "CTL-AD" | hsa-miR-877-3p, hsa-miR-30a-5p, hsa-miR-30c-5p, hsa-miR-181a-5p, hsa-miR-142-3p, hsa-miR-15b-5p | 6 |
| Elements in "CTL-MCI", "MCI-AD" and "CTL-AD" | hsa-miR-26b-5p, hsa-miR-335-5p, hsa-miR-92a-3p, hsa-miR-615-3p, hsa-miR-484, hsa-miR-16-5p, hsa-miR-17-5p, hsa-miR-218-5p, hsa-miR-24-3p, hsa-miR-124-3p, hsa-miR-93-5p, hsa-miR-193b-3p, hsa-miR-20a-5p | 13 |
| Element in "MCI-AD" and "CTL-AD" | hsa-miR-106b-5p | 1 |

**Supplementary Table S14.** The names, related modules and the number of miRNAs proposed as a biomarker by this study.
